# A revision of the genus *Beesia* (Ranunculaceae) as informed through integrative taxonomy, with description of a new species from Sichuan (China)

**DOI:** 10.3389/fpls.2025.1699952

**Published:** 2026-01-05

**Authors:** Andrey S. Erst, Elizaveta Yu. Mitrenina, Denis A. Krivenko, Tatyana V. Erst, Yulia V. Cheldysheva, Igor V. Gorbenko, Renata Borosova, Lian Lian, Yuan-Yuan Ling, Huan-Wen Peng, Jun Zhang, Shukherdorj Baasanmunkh, Hyeok Jae Choi, Ivan V. Tatanov, Alexander A. Kuznetsov, Mathew T. Sharples, Kun-Li Xiang

**Affiliations:** 1 Laboratory of Herbarium, Central Siberian Botanical Garden of the Siberian Branch of the Russian Academy of Sciences, Novosibirsk, Russia; 2 Department of Genetics and Cell Biology, Biological Institute, National Research Tomsk State University, Tomsk, Russia; 3 Department of Biodiversity and Biological Resources, Siberian Institute of Plant Physiology and Biochemistry of the Siberian Branch of the Russian Academy of Sciences, Irkutsk, Russia; 4 Laboratory of Molecular Phytopathology, Institute of Cytology and Genetics of the Siberian Branch of the Russian Academy of Sciences, Novosibirsk, Russia; 5 Laboratory of Biological Control of Phytophages and Phytopathogens, Siberian Federal Scientific Centre of Agrobiotechnologies of the Russian Academy of Sciences, Krasnoobsk, Russia; 6 Laboratory of Plant Genetic Engineering, Siberian Institute of Plant Physiology and Biochemistry of the Siberian Branch of the Russian Academy of Sciences, Irkutsk, Russia; 7 Herbarium, Royal Botanic Gardens, Kew, London, United Kingdom; 8 State Key Laboratory of Plant Diversity and Specialty Crops, Institute of Botany, Chinese Academy of Sciences, Beijing, China; 9 China National Botanical Garden, Beijing, China; 10 University of Chinese Academy of Sciences, Beijing, China; 11 Department of Biology and Chemistry, Changwon National University, Changwon, Republic of Korea; 12 Department of Herbarium of Higher Plants, Komarov Botanical Institute of the Russian Academy of Sciences, St. Petersburg, Russia; 13 Laboratory of the Herbarium, National Research Tomsk State University, Tomsk, Russia; 14 Department of Biological and Earth Sciences, Arkansas Tech University, Russellville, AR, United States

**Keywords:** *Beesia*, China, genome, karyotype, integrative taxonomy, new species, phylogeny, Ranunculaceae

## Abstract

**Introduction:**

Taxonomic diversity at different levels of organization is determined by different levels of biological diversity, including morphological, phylogenetic (genetic or/and genomic), and cytogenetic differences. However, focused studies utilizing a robust combination of such data are still in their infancy in plant systematics.

**Methods:**

Here, we integrate multiple forms of data to better understand species delimitation and taxonomy within the eastern Asiatic genus *Beesia* (Ranunculaceae), traditionally thought to include two species. Specifically, 26 morphological characters were measured, plastome regions were compared to reconstruct evolutionary relationships, and novel karyotype data were generated.

**Results:**

Morphological analyses parsed three species of *Beesia*, with little overlap expressed for most measured characters. Phylogenetic data reconstructed these species into monophyletic groups, and cytogenetic data uncovered distinct karyotypes between taxa. This integrative taxonomic approach enabled delimitation of a new cryptic species from Sichuan (China): *Beesia yangii* Erst & K.L. Xiang. In addition, suitable lectotypes for *Cimicifuga calthifolia* Maxim. ex Oliv., *Beesia cordata* Balf.f. & W.W.Sm., and *Beesia elongata* Hand.-Mazz.

**Discussion:**

Our study helps demonstrate how broader application of integrative approaches in systematic biology may both better inform recognition of previously undiscovered taxonomic diversity and better inform outlines for conservation of biodiversity. The newly uncovered species, *B. yangii*, is known from very few localities and is already of critical conservation concern.

## 1 Introduction

Species delimitation based on morphology can be challenging due to intraspecific variation among populations and limited differentiation between closely related species (Whittall et al., 2004; Lissambou et al., 2019; Wang et al., 2022). Accurate species delimitation and identification play a pivotal role in the assessment, monitoring, and conservation of biological diversity (Agapow et al., 2004; Carstens et al., 2013). Taxonomic study of biodiversity is particularly complicated by the presence of cryptic species, defined as two or more separate species that are incorrectly classified as a single species (Bickford et al., 2007; Fu et al., 2023). They may be barely distinguishable morphologically, albeit along different evolutionary trajectories (Struck et al., 2018; Fu et al., 2023). These hidden or unrecognized species represent a substantial part of undescribed biodiversity, posing challenges for realizing comprehensive taxonomy and robust biodiversity assessments and implementing well-informed conservation plans (Renner, 2020; Fu et al., 2023). To date, there remains no unified species concept relating to the angiosperm clade. This is because a “gray zone” frequently exists, in which boundaries between taxa become murky and may be difficult to define (De Queiroz, 2007; Bock et al., 2023). In contrast to oftentimes more simple cases of biological species resolution in the animal clade, the angiosperm clade in particular commonly exhibits, e.g., widespread apomixis and/or self pollination potentials, rampant hybridization across time and space (sometimes itself resulting in formation of novel lineages with new genetic architecture), phenotypic variation across often relatively large geographic ranges and/or phenotypic plasticity, and myriad forms of clonal reproduction in absence of the sexual process (Hörandl, 2010; Grossenbacher et al., 2017, Kremer and Hipp, 2020, Karbstein et al., 2024; Sharples and Manzitto-Tripp, 2024; Chambers et al., 2025, Erst et al., unpubl. data). These factors and others have led to multiple interpretations and categories of species delimitation in angiosperms, these often taxon-dependent.

Cryptic diversity can arise through different mechanisms (Liu et al., 2022; Li and Wiens, 2023), such as weak morphological differentiation among geographically proximate yet reproductively isolated populations (Sharples, in prep.), rapid radiation (Fior et al., 2013), convergent evolution driven by similar ecological pressures (Yeaman et al., 2016; Kinosian et al., 2020), hybridization (Aköz and Nordborg, 2019; Wróbel et al., 2024), and/or changes in chromosome structure (Erst et al., 2020; Mitrenina et al., 2024). An important approach to species identification and conservation of rare species is standard single-locus Sanger markers (Trivedi et al., 2016), which has also been useful in solving problems in taxonomy (Vargas et al., 2012; Ali et al., 2014). Standard single-locus Sanger markers can help to quickly and accurately identify species and has the potential to lead to the discovery of new species (Erst et al., 2022). However, standard single-locus Sanger markers are not always effective in systematic biology, especially when considering taxa that radiated recently or those that possess otherwise complex evolutionary histories (Percy et al., 2014; Yan et al., 2015). To address these challenges in part, a growing number of studies have involved multiple lines of evidence in species delineation, termed the integrative approach (Erst et al., 2020). This approach is especially effective for delimiting species with previously uncertain and unrecognized taxonomic status (e.g., Erst et al., 2020; Meudt et al., 2025; Wang et al., 2025).

**Figure 1:**
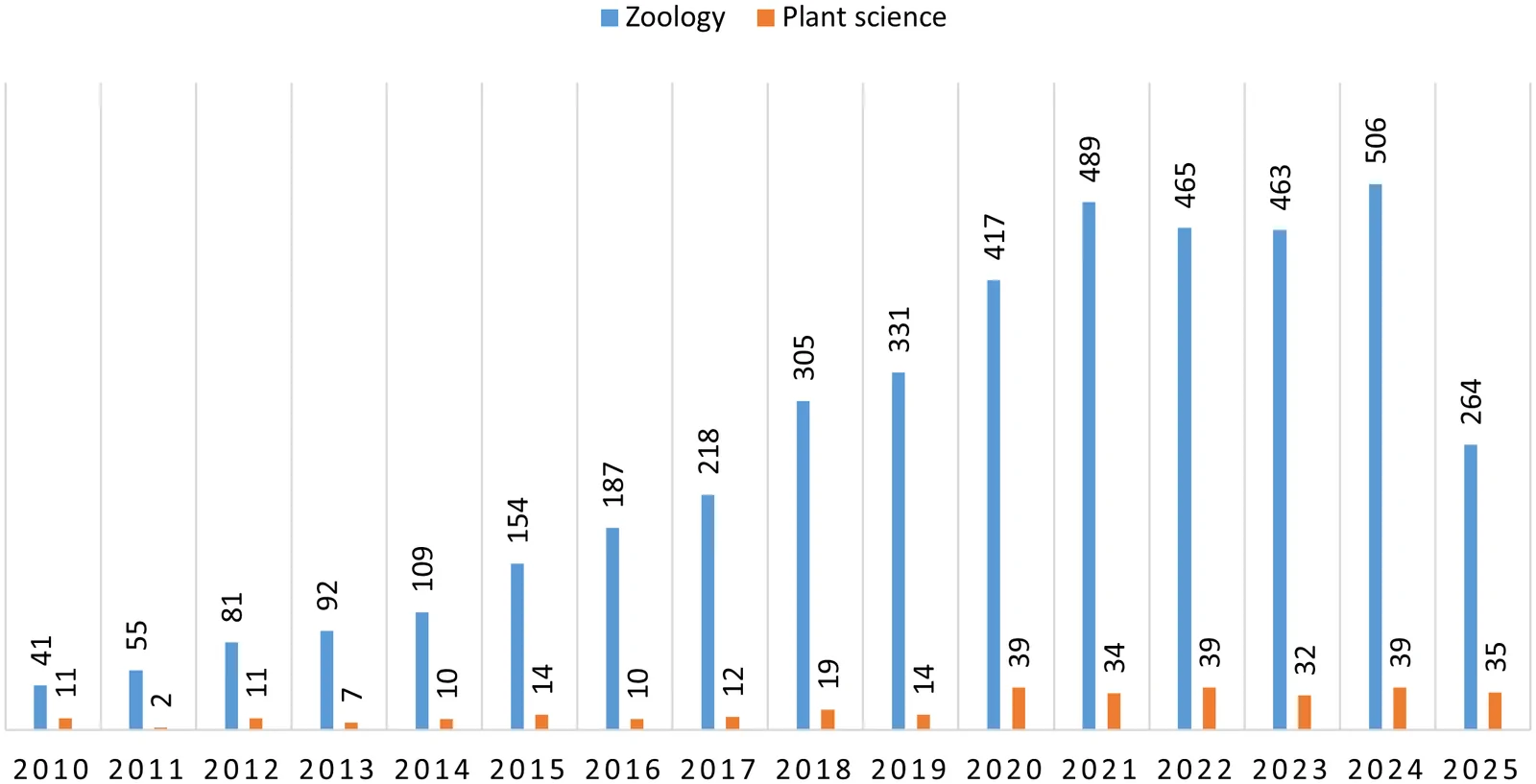
Diagram showing how many studies using “integrative taxonomy” in the title and/or abstract were conducted in zoology and plant science research from 2010 to 2025.

Searches conducted on the ISI Web of Science (www.webofscience.com/wos/alldb/basicsearch, last accessed on 7 August 2025) using “integrative taxonomy” in the title and/or abstract revealed that zoological studies using integrative taxonomy have rapidly increased recently, whereas botanical studies have rarely adopted this method (**Figure 1**). Recent taxonomic revisions and descriptions of new plant taxa increasingly rely on a combination of morphological traits and genetic data. However, plant systematics frequently encounters taxonomically complex groups (TCGs), in which overlapping evolutionary processes—such as hybridization, polyploidy, asexual reproduction, and/or incomplete reproductive isolation—obscure clear morphological and genetic distinctions (Ennos et al., 2005). These complexities often necessitate the integration of complementary methodologies, including cytogenetic, biochemical, and population genetic analyses, alongside traditional morphological and molecular approaches (Erst et al., 2020).

The Sino-Himalayan region is a temperate biodiversity hotspot with high levels of species endemism (Myers et al., 2000; Sun et al., 2017; Erst et al., 2023). It is also a modern center of distribution and diversification of elements of the temperate flora in the Northern Hemisphere (Peng et al., 2023; Sharples and Manzitto-Tripp, 2024). The Qinghai-Tibet Plateau (QTP), known as the “Roof of the World” and the “Asian water tower,” stands at a minimum elevation of 4,500 m high over a region of approximately 2.5 × 10^6^ km^2^ and consists of three subregions: the Plateau Platform, the Himalaya, and the Hengduan Mountains (Mao et al., 2021; Wu et al., 2022). At present, the QTP houses more than 1,500 genera and at least 12,000 species of vascular plants, of which more than 25% are endemic to this region (Wu and Wu, 1996; Wen et al., 2014). Although the biodiversity of the region has been extensively studied (e.g., Xue et al., 2021; Zhang et al., 2023), new taxa are still being described and the broader QTP is expected to harbor more undescribed taxa for decades to come.

The tribe Cimicifugeae Torr. & A.Gray belongs to the family Ranunculaceae Juss. and includes four genera and more than 49 species (Compton et al., 1998; Wang et al., 2005; Yuan and Yang, 2006; Handel-Mazzetti, 1922). This tribe contains *Anemonopsis* Siebold & Zucc. (one species), *Actaea* L. (32 species), *Eranthis* Salisb. (14 species), and *Beesia* Balf.f. & W.W.Sm. (two species), and the tribe as a whole exhibits a circumboreal distribution (Compton and Culham, 2002; Ling et al., 2023). Two genera are restricted regional endemics, i.e., *Anemonopsis* is known only from Japan, and *Beesia* occurs only in China, India, and Northern Myanmar (Wang et al., 2001; Bhaumik and Krishna, 2024; Kostikova et al., 2024; Liangqian and Tamura, 2001). The genus *Beesia* was originally described by Balfour and Smith (1915), and the new taxon was thought to be closely related to the Japanese genus *Glaucidium* Siebold & Zucc. and the Japanese-American genus *Hydrastis* J.Ellis. In the same publication, the authors also described a new species: *Beesia cordata* Balf.f. & W.W.Sm. from Northern Burma (Myanmar). Before the introduction of the genus *Beesia*, *B. cordata* had already been described by Daniel Oliver based on material collected by Karl Johann Maximowicz (in Hooker’s *Icones Plantarum*: Oliver, 1888) as *Cimicifuga calthifolia* Maxim. ex Oliv.; therefore, *Beesia cordata* is currently recognized as a synonym of *B. calthifolia* (Maxim. ex Oliv.) Balf.f. & W.W.Sm (Balfour and Smith, 1924; Tiwari and Mao, 2016). The other currently recognized species of *Beesia* is *B. deltophylla* C.Y.Wu. Within its range, *B. calthifolia* is represented by populations that exhibit significant morphological differences, and furthermore, some differences in karyotype structure and ploidy have been noted by previous authors within *B. calthifolia* (Yang et al., 1995). Recent molecular phylogenetic studies have shown multiple clades among populations of *B. calthifolia* populations depending on the altitude and climatic conditions of a habitat (Peng et al., 2023).

While the tribe Cimicifugeae has been studied in detail by many authors utilizing individual methods, an integrative approach has not been applied to any members of the group until now. In the present study, we explore multiple levels of diversity of the genus *Beesia* to forward a taxonomic revision of this group. We utilize an integrative approach involving morphological, phylogenetic, and cytogenetic analyses to evaluate species delimitation and to study the taxonomy of this group. A combination of the above approaches has been successfully applied in previous studies of related taxa (Erst et al., 2020, Erst et al. in review, *Eranthis*), and other studies have also taken an integrative approach to taxonomy, using different sets of methods (Meudt et al., 2025; Wang et al., 2025, etc.). Based on these multiple lines of evidence, a revised treatment of species boundaries and a revision within this genus are presented. Three suitable lectotypes for *Cimicifuga calthifolia* Maxim. ex Oliv., *Beesia cordata* Balf.f. & W.W.Sm., and *Beesia elongata* Hand.-Mazz. were selected in accordance with articles 9.3 and 9.12 of the ICN (Turland et al., 2025).

## 2 Materials and methods

### 2.1 Plant material

More than 100 herbarium specimens from numerous localities were freshly collected during field investigations in China (Sichuan Province and Tibet Autonomous Region) during the 2024–2025 field seasons. Dried and preserved specimens were deposited in the NS (I.M. Krasnoborov Herbarium of the Central Siberian Botanical Garden of the Siberian Branch of the Russian Academy of Sciences, Novosibirsk, Russia), IRK (Herbarium of the Siberian Institute of Plant Physiology and Biochemistry of the Siberian Branch of the Russian Academy of Sciences, Irkutsk, Russia), and LE (Komarov Botanical Institute of the Russian Academy of Sciences, St. Petersburg, Russia) Herbaria (herbarium abbreviations according to Thiers, 2018) (**Supplementary Table S1**). A review of existing herbarium materials was undertaken through digitized images or physically in Herbaria at ARUN, BM, CAL, E, K, KUN, LE, NS, NY, P, PE, TI, US, and WU. Drawings of *Beesia yangii* sp. nov. (1), *B. calthifolia* (2), and *B. deltophylla* (3) were created based on images of type (1: NS0000490–NS0000492) and non-type specimens (2: NS0003871, NS0003872; 3: NS0033868–NS0033870). Maps of records were prepared with R (v4.4.1; R Core Team, 2024).

### 2.2 Morphological analysis

A total of 26 different morphological traits were quantitatively measured or otherwise assessed from the above herbarium materials (**Table 1**). After measurement or assessment of traits, analysis of morphological data was conducted using R with the following packages (v4.4.1; R Core Team, 2024). In case of unavailable or otherwise missing data values, missing-measurement imputation was performed with the missMDA package (v1.19; Audigier et al., 2017). To determine if morphospecies clustered or not, principal component analysis was performed with the help of the FactoMineR package (v2.11; Lê et al., 2008). Visualization of the morphological data was conducted using packages factoextra (v1.0.7; Kassambara and Mundt, 2020), ggplot2 (v3.5.1; Wickham, 2016), and ggpubr (v0.6.0; Kassambara, 2023). For assessment of the specimens’ data distribution, *ad hoc* (Kruskal–Wallis) and *post-hoc* (Mann–Whitney–Wilcoxon) tests were carried out with the rstatix package (v0.7.2; Kassambara, 2023).

**Table 1:** Morphological differences among species of *Beesia.*

№	Characteristic	*B. deltophylla*	*B. calthifolia*	*B. yangii*
1	Plant height, cm*	14–58	14–87.5	40.5–74.7
–	Rhizome color	Purple or pink	Yellow or light brown	Purple or pink
2	Rhizome length, cm*	~10	~15	~10
3	Rhizome diameter, cm*	0.5–0.7	0.3–0.9	0.4–1.1
–	Scape trichome type	Unicellular lageniform	Unicellular lageniform	Unicellular lageniform
4	Scape pubescence in upper part (+/–)*	1	1	1
5	Scape pubescence in lower part (+/–)*	1	1	1
–	Leaf blade shape	Cordate-triangular	Reniform	Cordate
–	Leaf blade color	Dark green	Light green	Dark green
–	Leaf blade vein color	Light veins	Inconspicuous	Light veins
–	Leaf blade texture	Glossy	matte	Glossy
–	Leaf blade apex	Acuminate	Rounded to acuminate	Acuminate
6	Leaf petiole length, cm*	5.5–15.0	5.6–26.5	8.4–31.15
7	Leaf blade length, cm*	2.1–8.4	2.4–9.5	1.9–13.5
8	Leaf blade width, cm*	1.9–4.5	4.0–13.6	3.0–12.0
–	Edge of the leaf blade	Crenulate-mucronate	Crenulate-mucronate	Crenulate-mucronate
–	Shape of leaf teeth	Crenately lobed, each lobe with mucronate apex, some lobes doubly lobed	Toothed, widely crenate with long mucro at apex, sometimes off-center	Toothed, narrowly triangular with long sharp mucro
9	Number of teeth*	14–51	22–179	26–120
10	Length of the tooth at the base of the leaf, mm*	1.66–5.62	0.83–1.86	1.05–2.98
11	Width of the tooth at the base of the leaf, mm*	2.58–8.31	1.9–5.84	0.98–5.31
12	Length of the mucro at the base of the leaf, mm*	0.48–1.1	1.29–0.65	0.5–1.0
13	Length of the tooth middle part of the leaf blade, mm*	1.46–4.36	0.78–1.67	1.23–2.56
14	Width of the tooth middle part of the leaf blade, mm*	3.49–7.97	1.87–5.90	1.45–6.97
15	Length of the mucro middle part of the leaf blade, mm*	0.43–1.0	0.31–0.69	0.45–0.94
16	Length of the mucro at the apex the leaf blade, mm*	2.79–11.99	2.05–9.72	2.54–14.46
17	Width of the mucro at the apex the leaf blade, mm*	2.62–7.11	2.13–11.49	0.85–6.93
18	Length of the mucro at the apex of the leaf, mm*	0.3–1.2	0.31–0.71	0.33–1.4
19	Flower diameter, cm*	0.6–0.9	0.8–1.1	1.5–2.2
20	Length of peduncle, cm*	0.4–1.0	0.3–0.8	0.5–1.0
	Sepal shape	Wide elliptic	Wide elliptic	Narrow elliptic
	Sepal color	White or pink	White or pink	White or white-green
	Sepals/stamens length ratio	>/=	<	>/=
21	Sepal length, cm*	0.4–0.5	0.4–0.6	0.5–0.9
22	Sepal width, cm*	0.2–0.25	0.15–0.20	0.2–0.4
23	Stamens length, cm*	0.4–0.6	0.30–0.31	0.4–0.8
24	Number of flowers in an inflorescence*	1–10	3–20	3–26
25	Follicle length, cm*	0.6–1.2	0.6–1.3	0.7–1.8
26	Persistent style length, сm*	0.1–0.2	0.1–0.3	0.1–0.2

An asterisk and numbers indicates traits used in numerical analysis.

The numbers indicate the characters that were used in the morphological analysis.

### 2.3 Molecular analysis

#### 2.3.1 Taxon sampling and sample sequencing

A total of 27 individuals of *Beesia*, representing all three species (including the new species), were included in molecular phylogenetic analysis. One species of *Actaea*, one species of *Anemonopsis*, and one species of *Eranthis*, covering all three other genera of Cimicifugeae, were also included. On the basis of other studies (e.g., Wang et al., 2009, 2016), three species—*Ranunculus ternatus* Thunb., *Aquilegia kansuensis* (Brühl) Erst, and *Caltha palustris* L.—were selected as outgroups, and most ingroup samples were obtained from Peng et al. (2023) (**Supplementary Table S2**). For three newly sequenced individuals, genomic DNA of samples was extracted from silica gel-dried leaves or herbarium specimens and was purified using the Tiangen Isolation/Extraction/Purification Kit (Tiangen Biotech [Beijing] Co., Ltd.). Genomic libraries targeting fragment lengths of 350 base pairs (bp) (via sonication) were prepared off-site for sequencing on the Illumina HiSeq X-Ten platform to assemble plastomes from the above samples based upon paired-end reads with lengths of 150 bp (Novogene [Beijing] Co., Ltd.). Approximately 10 GB of raw sequencing data was newly generated for each sample. To obtain high-quality genome sequences, raw reads were processed using Fastp v0.19.7 (Chen et al., 2018) to remove adapter sequences and low-quality reads. Peng et al. (2023) did not include nuclear data, and thus our new datasets likewise only represented plastid sequences.

#### 2.3.2 Data assembly and annotation

The plastome for newly generated samples was assembled *de novo* in GetOrganelle v1.7.6.1 (Jin et al., 2020) with default parameters. The obtained plastid contigs were further visualized and scaffolded to complete circular genomes in Bandage v0.8.1 (Suyama et al., 2006). Highly accurate annotation of organelle genomes was performed by using the Organellar Genome GeSeq tool (Tillich et al., 2017) with subsequent manual correction. Two plastomes from *Beesia deltophylla* (GenBank accession No. NC_072729) and *B. calthifolia* (NC_041531) were used as reference sequences. We employed OGDRAW v1.3.1 (Greiner et al., 2019) to visualize the circular plastome map. Amino acid sequences of 78 plastid protein-coding-gene regions were extracted for phylogenetic analysis, and each was aligned in MAFFT v7 (Katoh and Toh, 2010). Finally, the corresponding three-codon-aligned DNA sequences were generated from the amino acid alignments using PAL2NAL v14 (Suyama et al., 2006).

#### 2.3.3 Phylogenetic analysis

Phylogenetic analyses of ingroup and outgroup samples were conducted based on concatenated DNA sequences from the coding regions of 78 protein-coding genes. Maximum likelihood (ML) analysis was performed using RAxML v8.2.11 (Stamatakis, 2014) with 1,000 replicates under the GTRGAMMA model.

### 2.4 Cytogenetic analysis

Cytogenetic analyses were performed on seven populations. Somatic chromosomes of the three species of *Beesia* were prepared from root meristems. Rhizomes were germinated at room temperature for 1–3 months. Newly formed 1–2-cm-long roots were excised and pretreated with a 0.5% colchicine solution for 3–4 h. Roots were then fixed in a mixture of 96% ethanol and glacial acetic acid (3:1). Chromosomes were stained with 1% aceto-hematoxylin and then subjected to morphometric analyses. Staining and karyotyping were performed according to standard protocols (Smirnov, 1968; Mitrenina et al., 2021). Mitotic metaphase chromosome plates were examined under an Axiostar microscope (Carl Zeiss, Munich, Germany) and photographed using an Axio Imager A.1 microscope (Carl Zeiss, Germany) equipped with an AxioCam MRc5 CCD camera (Carl Zeiss, Germany) at 1,000× magnification by means of AxioVision 4.7 software (Carl Zeiss, Munich, Germany) in the Laboratory for Ecology, Genetics and Environmental Protection (EcoGene) of the National Research Tomsk State University (Tomsk, Russia). KaryoType 2.0 software (Altınordu et al., 2016) was employed for karyotyping, and Adobe Photoshop CS5 (Adobe Systems, USA) and Inkscape 0.92 (USA) for image editing.

Symbols used to describe the karyotypes matched those defined elsewhere (Levan et al., 1964): m = a median centromeric chromosome with an arm ratio (r) of 1.0–1.7 (metacentric chromosome); sm = a submedian centromeric chromosome with an arm ratio of 1.7–3.0 (submetacentric chromosome); st = a subterminal centromeric chromosome with arm ratio 3.0–7.0 (subtelocentric chromosome); t = a terminal centromeric chromosome with arm ratio 7.0 or more (telocentric chromosome); T = a chromosome without an obvious short arm. Mean values of the arm ratio (r) and relative chromosome length (RL) for each chromosome pair and total haploid length (THL) were determined. In addition, we calculated the coefficient of variation of chromosome length (CV_CL_) (Paszko, 2006), the coefficient of variation of the centromeric index (CV_CI_) (Paszko, 2006), and mean centromeric asymmetry (M_CA_) (Peruzzi and Eroğlu, 2013).

### 2.5 Conservation assessment

To assess the conservation status of species of *Beesia*, we utilized IUCN Red List criteria for placing taxa into one of six categories: critically endangered (CR), endangered (EN), vulnerable (VU), near threatened (NT), least concern (LC), or data deficient (DD), according to current guidelines and criteria defined by the IUCN Standards and Petitions Committee (2024). In general, there are five evaluation criteria defined by the IUCN Standards and Petitions Committee (2024): population size reduction (criterion A); geographic range size fragmentation, few locations, range decline, or population fluctuations (B); small and declining population size and fragmentation, fluctuations, or few subpopulations (C); very small population or very restricted distribution (D); and quantitative analysis of extinction risk (E). Among these, criterion B—severity of range fragmentation—is suitable for estimating conservation status, even if data are limited and the distribution of a taxon is known from only few georeferenced records. In this study, we used criterion B because distribution records of the three species of *Beesia* have been relatively well compiled. Specifically, these distribution records were extracted from herbarium labels at the above institutions, including those from our own collections and associated published research. Subcriteria B1 (Extent of Occurrence, EOO) and B2 (Area of Occupancy, AOO) were estimated with the ConR package (Dauby et al., 2017) in R (R Core Team, 2024). The minimum AOO was estimated based on a user-defined grid cell of 2 km^2^ as recommended by the IUCN (2024). In particular, for the AOO and EOO, the ConR package provides the ability to incorporate the unique number of occurrences, the number of subpopulations, and locations of each taxon into analyses.

## 3 Results

### 3.1 Morphological analysis

**Figure 2:**
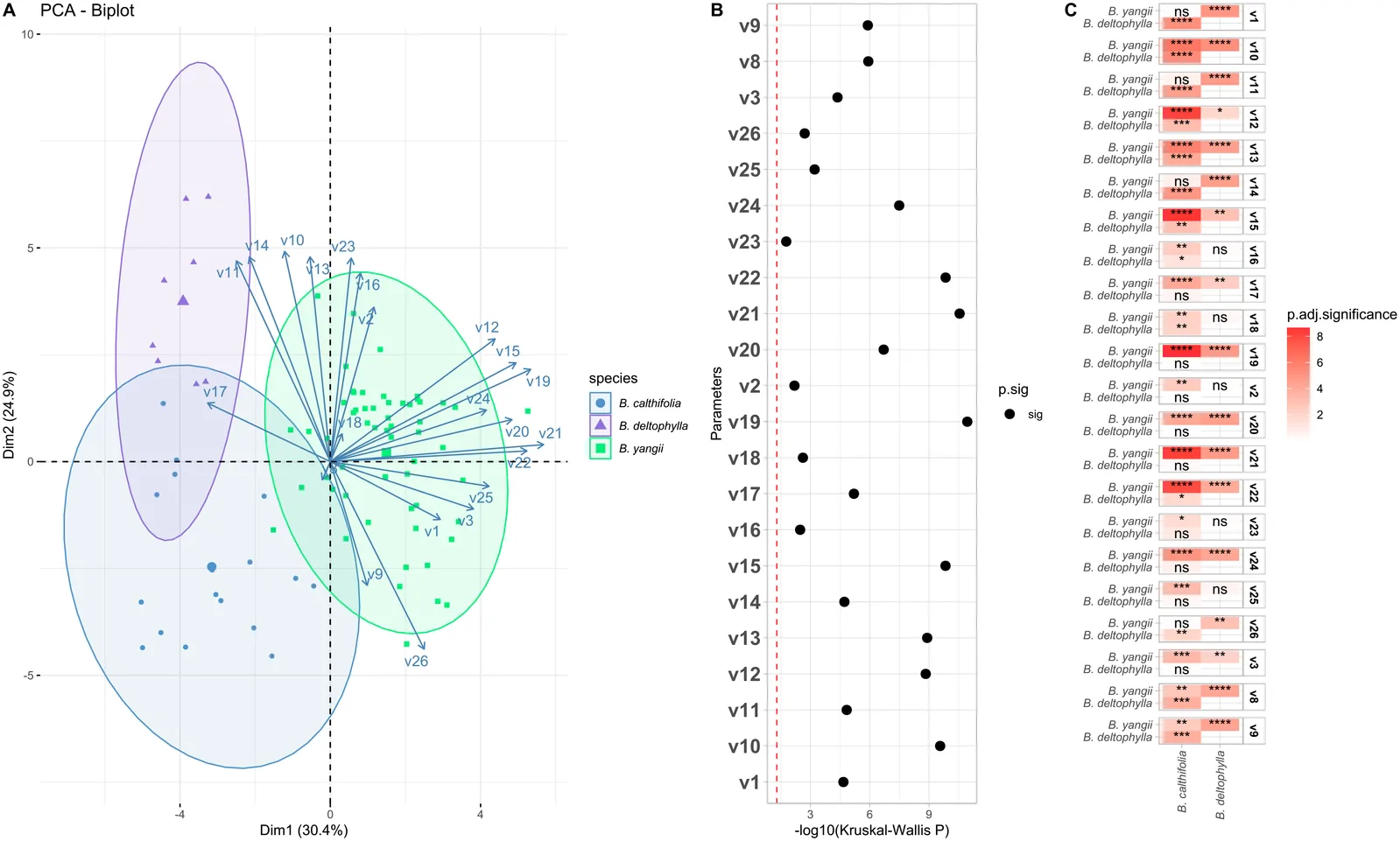
Results of principal component analysis. **(A)** Principal component analysis biplot of measured parameters between different species of *Beesia*. **(B)** Dot plot of the significance of the Kruskal–Wallis test [p-value: −log_10_(P)] of measured parameters. The vertical red dashed line represents the p = 0.05 threshold. **(C)** A heatmap of adjusted p-values (Benjamini–Hochberg correction) from the interspecies pairwise Mann–Whitney–Wilcoxon test of measured parameters. Asterisks represent adjusted p-values (significance): ns, not significant, *p < 0.05, **p < 0.01, ***p < 0.001, ****p < 0.00001.

Results of principal component analysis and the significance of interspecies differences suggested recognition of three taxa of *Beesia* (**Figure 2**). The largest contribution to the first two principal components (PCs) was made by variables 19 (flower diameter) and 21 (sepal length) (**Table 1**). Data areas of *B. calthifolia* and *B. yangii* sp. nov. were separated with a small intersection. There was separation across gradients of all tested parameter values except for parameters 11 (width of the tooth at the base of the leaf), 14 (width of the tooth middle part of the leaf blade), and 26 (persistent style length) (**Table 1**). The data area of *B. deltophylla* was found to be completely separated from the area of *B. yangii* but exhibited an intersection with the area of *B. calthifolia* (**Figure 2A**). Categorical variables 4 (scape pubescence in upper part) and 5 (scape pubescence in lower part) were identical among the examined species.

The Kruskal–Wallis test revealed that all the variables have statistically significant interspecies differences (**Figure 2B**). Pairwise interspecies comparisons of measured parameters are given in **Figure 2C**.

### 3.2 Molecular analysis

**Figure 3:**
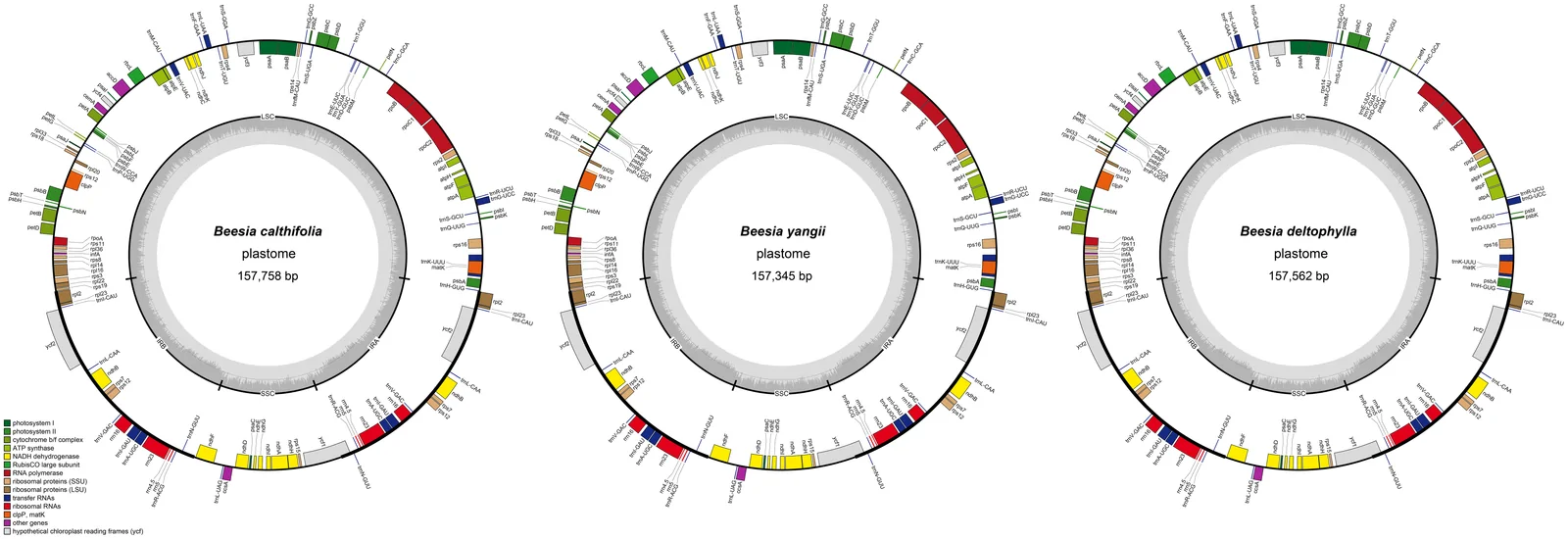
Plastome maps of species of *Beesia*.

The plastomes of species of *Beesia* that were assembled in this study exhibited complete sizes from 157,345 bp in *B. yangii* sp. nov. to 157,758 bp in *B. calthifolia* (**Figure 3**). All plastomes exhibited the quadripartite structure typical of many plastomes, which comprises a pair of IRs (inverted repeats) (26,516–26,584 bp) separated by an LSC (long single copy) region (86,971–87,252 bp) and an SSC (small single copy) region (17,206–17,474 bp; **Figure 3**). We identified 130 predicted functional genes and one pseudogene (*ψycf1* in IR_B_/SSC) for the plastomes of *Beesia*. These genes were summarized as 112 unique genes (with 18 of them repeated) including 78 protein-coding genes, 30 transfer RNA (tRNA) genes, and 4 ribosomal RNA (rRNA) genes (**Figure 3**).

**Figure 4:**
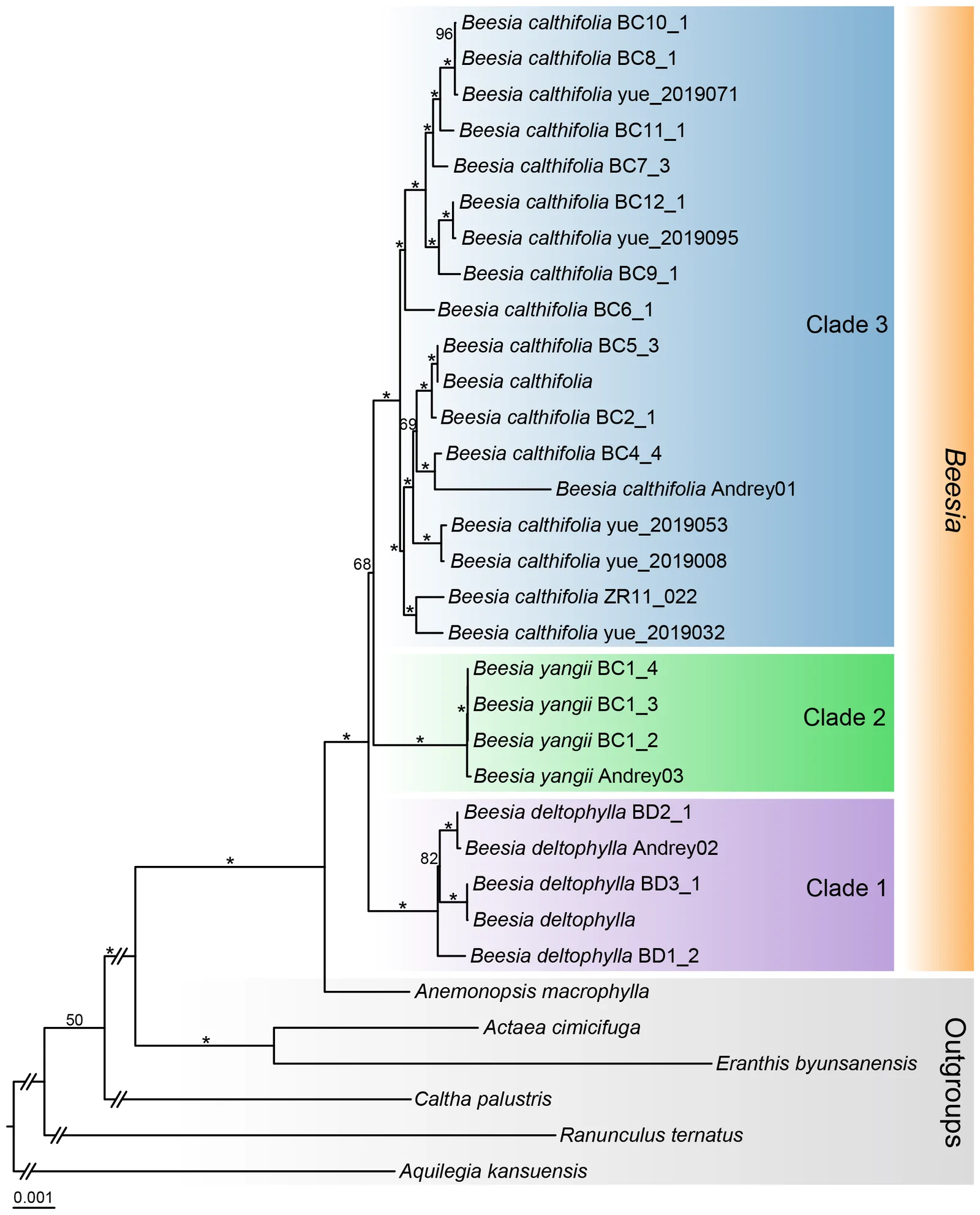
The ML tree inferred from the combined 78 plastid protein-coding-gene regions with numbers above branches representing bootstrap values (BS > 50%). The BC1–BC12 and BD1–3 populations correspond to the BC1–12 and BD1–3 populations in Peng et al. (2023). “*” denotes BS = 100%.

The DNA supermatrix comprised 70,009 aligned nucleotides from the 78 protein-coding genes. Maximum likelihood analysis indicated that Cimicifugeae is strongly supported as monophyletic (bootstrap support [BS] = 100%) (**Figure 4**). Within Cimicifugeae, *Actaea* clustered with *Eranthis* (BS = 100%). *Beesia* was reconstructed as a monophyletic group with strong support (BS = 100%) and as sister taxon to *Anemonopsis* (BS = 100%). In *Beesia*, three major clades (1, 2, and 3) were identified, corresponding to the species *B. deltophylla* (BS = 100%), *B. yangii* (BS = 100%), and *B. calthifolia* (BS = 100%), respectively. *Beesia yangii* and *B. calthifolia* formed a clade (BS = 68%) that was reconstructed as sister to *B. deltophylla* (BS = 100%).

### 3.3 Cytogenetic analysis

**Figure 5:**
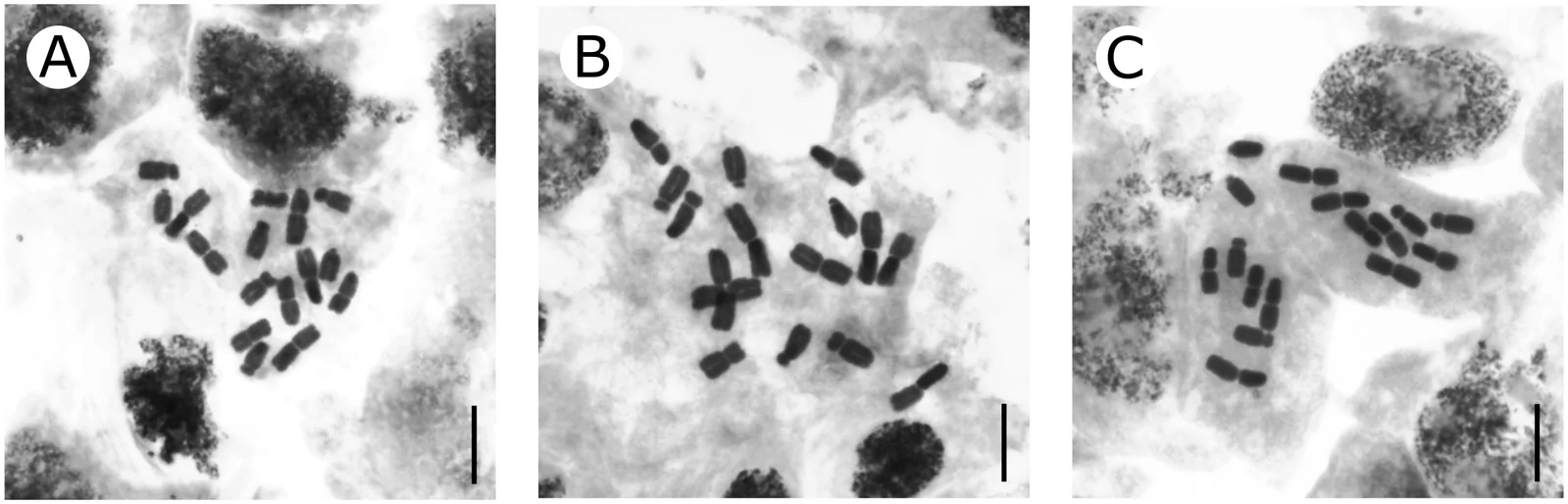
Mitotic metaphase chromosomes of the three species of *Beesia*. **(A)** *B*. *calthifolia*, 2*n* = 16. **(B)** *B*. *deltophylla*, 2*n* = 16. **(C)** *B*. *yangii*, 2*n* = 16. Scale bars = 10 µm.

Karyotypes of *B. calthifolia* (Sichuan Province), *B. deltophylla* (Mêdog County), and *B. yangii* sp. nov. (Sichuan Province) were newly generated. All the studied specimens were found to be diploid with *2n* = 16 (**Figure 5**). Karyometric analysis was performed on chromosomes with different degrees of compaction (estimated using total haploid length) for more accurate determination of karyotype formulas. The mean values (**Table 2**) were obtained from measurements in metaphase plates having similar degrees of chromosome compaction, usually the highest among those available on a cytological slide. Intrachromosomal and interchromosomal asymmetry and heterogeneity of the centromeric index were similar among the three examined species of *Beesia* (**Table 2**).

**Table 2:** Karyomorphological parameters for the three species of *Beesia*.

Species	Pair	CL, µm	r	RL, %	Type	THL	MC_A_	CV_CL_	CV_CI_
*B. calthifolia*	1	8.57 ± 0.13	1.21 ± 0.05	8.21	msat	52.16 ± 1.58	28.18 ± 0.41	19.73 ± 0.80	36.27 ± 1.37
	2	7.95 ± 0.39	1.08 ± 0.05	7.62	m				
	3	7.36 ± 0.26	1.08 ± 0.04	7.06	msat				
	4	6.48 ± 0.39	1.24 ± 0.08	6.21	m				
	5	5.69 ± 0.33	1.76 ± 0.09	5.45	sm				
	6	5.68 ± 0.35	2.30 ± 0.17	5.45	sm				
	7	5.63 ± 0.31	3.37 ± 0.35	5.39	st				
	8	4.81 ± 0.26	7.79 ± 0.53	4.61	t				
*B. deltophylla*	1	9.47 ± 0.66	1.03 ± 0.02	8.12	m	58.29 ± 2.92	26.72 ± 1.12	20.64 ± 1.19	36.27 ± 1.37
	2	9.29 ± 0.49	1.14 ± 0.06	7.97	m				
	3	8.16 ± 0.59	1.06 ± 0.04	7.00	m				
	4	7.31 ± 0.48	1.26 ± 0.05	6.27	m				
	5	6.29 ± 0.41	1.52 ± 0.05	5.40	m				
	6	6.39 ± 0.14	2.58 ± 0.22	5.48	sm				
	7	5.90 ± 0.38	3.67 ± 0.60	5.06	st				
	8	5.48 ± 0.31	5.90 ± 0.65	4.70	stsat				
*B. yangii*	1	7.24 ± 0.23	1.20 ± 0.05	8.06	m	44.92 ± 0.35	25.80 ± 1.23	17.99 ± 1.60	34.86 ± 1.43
	2	6.73 ± 0.37	1.21 ± 0.05	7.49	m				
	3	6.31 ± 0.19	1.06 ± 0.02	7.02	msat				
	4	5.42 ± 0.16	1.27 ± 0.04	6.03	m				
	5	5.12 ± 0.08	1.10 ± 0.05	5.70	msat				
	6	4.97 ± 0.14	2.43 ± 0.15	5.53	sm				
	7	4.87 ± 0.18	3.45 ± 0.26	5.42	st				
	8	4.27 ± 0.27	6.23 ± 0.61	4.75	st				

Pair, chromosome pair; CL, chromosome length (mean ± SD); r, arm ratio (mean ± SD); RL, relative chromosome length; m, metacentric chromosome; sm, submetacentric chromosome; st, subtelocentric chromosome; t, telocentric chromosome; sat, chromosome with a satellite or secondary constriction; THL, total haploid length (mean ± SD); CVCL, coefficient of variation of chromosome length (mean ± SD); MCA, mean centromeric asymmetry (mean ± SD); CVCI, coefficient of variation of centromeric index (mean ± SD).

**Figure 6:**
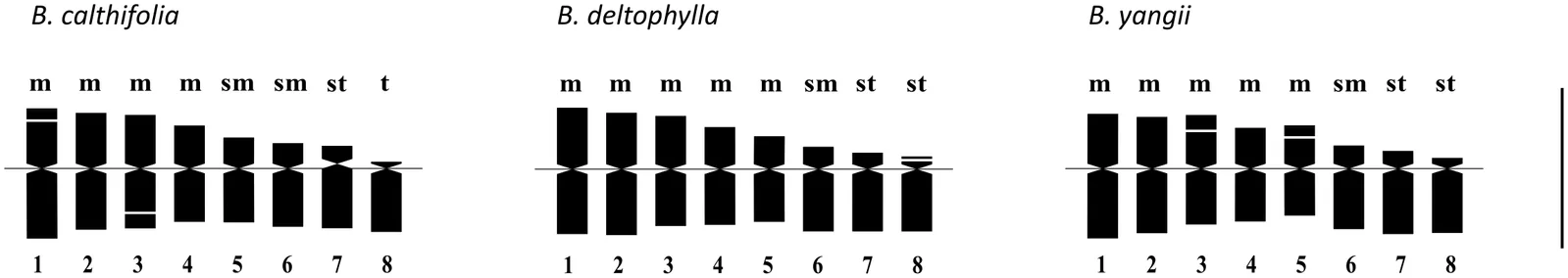
Haploid idiograms for the three species of *Beesia*. m—metacentric chromosome; sm—submetacentric chromosome; st—subtelocentric chromosome; t—telocentric chromosome; 1–8—chromosome pairs. Scale bar = 10 µm.

Karyotype structure of the studied specimens of *B. calthifolia* corresponds to the formula 2*n* = 4m + 4m^sat^ + 4sm + 2st + 2t (**Figure 6**). Metacentric chromosomes differed in length (relative length varies on average from 6.21% to 8.21%), with the second and third pairs slightly more symmetrical than the first and fourth pairs (**Table 2**). The first and third longest pairs, as a rule, bore secondary constrictions. The fifth and sixth pairs of chromosomes were submetacentric, had similar lengths (relative length is on average 5.45%), but could be well differentiated by the arm ratio. This ratio of the fifth pair was close to a borderline value, and the type of these chromosomes is metacentric according to Levan et al. (1964). The seventh pair was close in length to the submetacentrics (relative length on average 5.39%) but is subtelocentric. The eighth pair was the shortest (relative length 4.61% on average) and is telocentric.

The karyotype formula in the studied specimens of *B. deltophylla* was 2*n* = 10m + 2sm + 2st + 2st^sat^ (**Figure 6**). Chromosomes of pairs 1 and 2 were similar in length (relative length of 8.12% and 7.97%, respectively, on average) but usually could be differentiated by the arm ratio (**Table 2**). Pairs 3, 4, and 5 differed both in length and in the arm ratio. The shortest of them was the most asymmetric. Unequal-armed chromosomes were represented by a pair of submetacentrics and two pairs of subtelocentrics of gradually decreasing length. Chromosomes of the eighth pair were formally classified as the subtelocentric type according to the average arm ratio, although on some metaphase plates, this parameter was very close to a value borderline with the telocentric type of chromosomes: 7.0. This pair of chromosomes was found to have small satellites on the short arm.

Karyotype structure of the studied specimens of *B. yangii* is expressed by the formula 2*n* = 6m + 4m^sat^ + 2sm + 4st (**Figure 6**). Pairs of metacentric chromosomes differed well in length and in the arm ratio. Chromosomes of pairs 3 and 5 bore secondary constrictions. The sixth and seventh pairs were similar in length but differed in the arm ratio: one is submetacentric, and the other is subtelocentric. The eighth pair was the shortest and is subtelocentric.

## 4 Discussion

### 4.1 Cryptic diversity of *Beesia* taxa in East Asian region and description of new species

**Figure 7:**
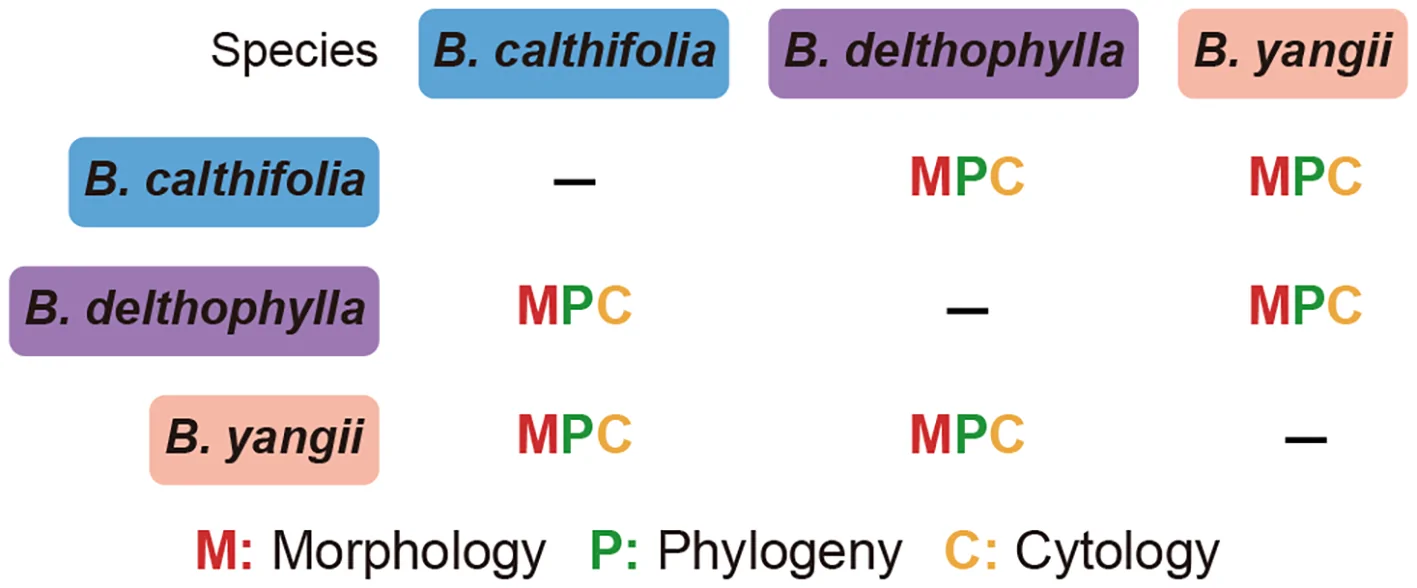
Schematic illustration of species delineation within *Beesia* and various sources of information used to distinguish species.

In this study, a framework for species delimitation in *Beesia* has been formulated, as derived through utilization of molecular, morphological, and cytogenetic data across populations of its species. Through these data using the integrative taxonomic approach, a new species of the genus *Beesia* has been uncovered, and lectotypes were selected (see taxonomic treatment below). All approaches, including genomic, cytogenetic, and morphological analyses, support the description of the new species and allow for comprehensive comparison of differences among the three species (**Figure 7**). Our phylogenetic analyses strongly supported *Beesia* as monophyletic (**Figure 4**), in agreement with previous studies (Peng et al., 2023).

Recent studies on the biogeographic histories of *Megacodon* (Hemsl.) Harry Sm. and *Beesia* by means of various data (ddRAD-seq, chloroplast genomes, molecular fragments, and morphological data) to identify similarities in evolutionary histories between the two genera showed that *Beesia* and *Megacodon* began to diverge from the late Miocene onward, with ancient allopatry at lower elevations resulting in narrow species ranges or narrow distributions of relict populations (Peng et al., 2023). Such processes may help explain why the two sister species of *Beesia* (*B. calthifolia* and newly described *B. yangii*) were reconstructed along distinct evolutionary trajectories. Mantel tests between genetic distance and climatic, elevational, or geographic distance revealed an isolation-by-distance pattern in *Beesia* and *Megacodon stylophorus* (C.B.Clarke) Harry Sm (Peng et al., 2023). *Beesia calthifolia* exhibited a genetic divergence pattern along an elevation gradient. Furthermore, we performed morphological measurements on *B. calthifolia* and found that different elevational groups have distinct leaf shapes (Peng et al., 2023). Nevertheless, the phylogeny of *Beesia* in this work uncovered genetic heterogeneity within populations of *B. calthifolia*. The plastome, cytogenetic, and morphological data obtained here allowed for a more robust taxonomic assessment compared with prior work in the group and supported the monophyly of the new species from Sichuan Province (China) and of two related taxa.

**Figure 8:**
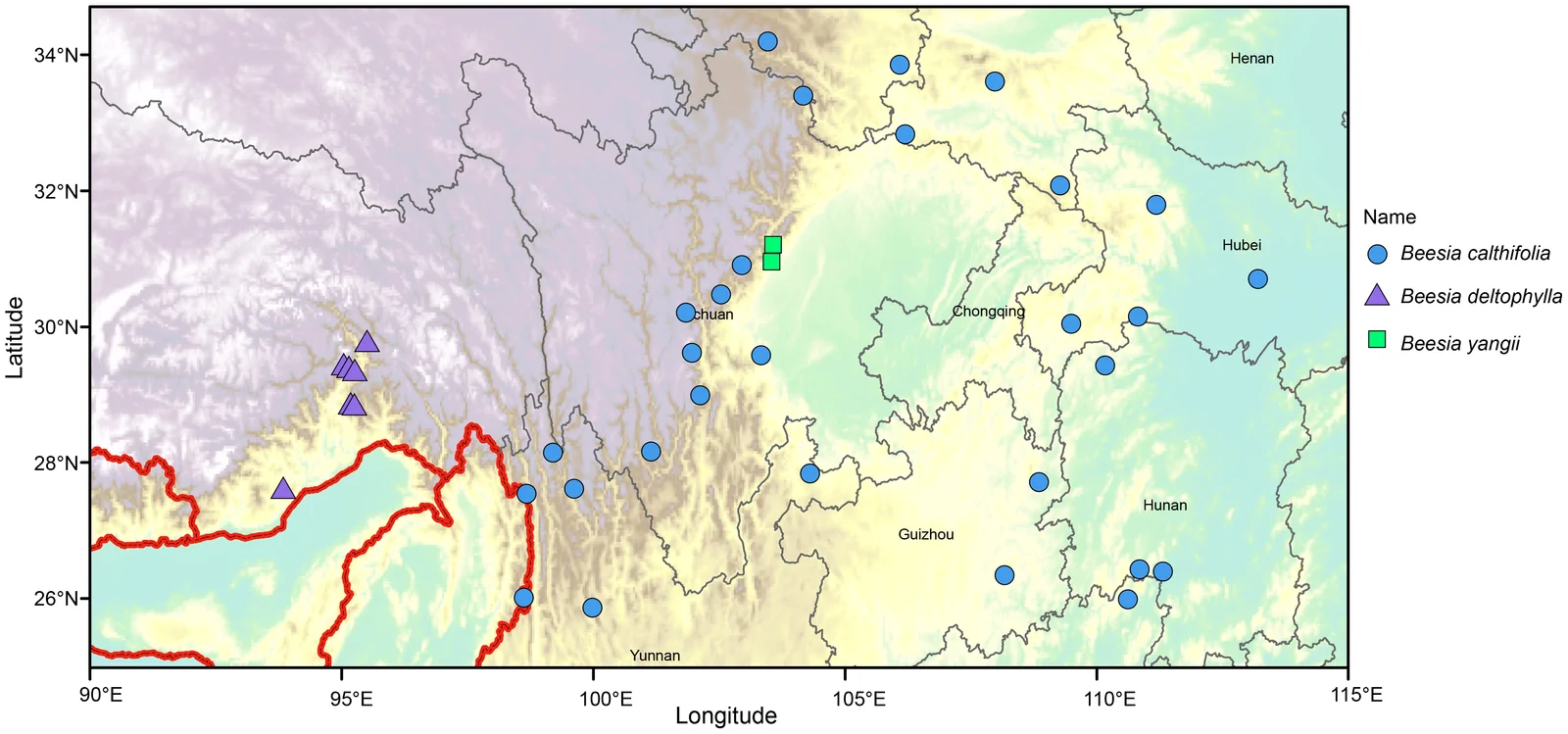
Distribution map of known localities of occurrence for species of *Beesia*.

For many genera, and especially for endemic and rare taxa poorly represented in herbarium collections, morphological identification criteria are poorly developed (e.g., many representatives of the Ranunculaceae). This work presents new morphological (and geographical) comparisons that are suitable for identifying species of the genus *Beesia* (see key below). The newly identified trait states, such as those involving leaf texture, the presence of colored leaf veins, shape and size of leaf teeth, width and length of sepals, and the ratio of stamens’ and sepals’ lengths, may now be utilized by field and herbarium botanists alike. *Beesia calthifolia* differs from other species of the genus by its reniform basal leaves with a light-green (with inconspicuous veins) dull surface, the apex rounded or acuminate, the blade with toothed edges (these teeth sometimes off-center) that are widely crenate with a long mucro at the apex, a yellow or light brown rhizome, and sepals that are shorter than the stamens. In contrast, the other two species produce basal leaves that are cordate or cordate-triangular, with a dark-green [with lighter-green veins] glossy surface and an acuminate apex, the margins toothed to crenately lobed, the teeth narrowly triangular with a long sharp mucro or each lobe with mucronate apical teeth, some lobes doubly lobed, a pink or purple rhizome, and sepals that are longer than the stamens. The new species *B. yangii* bears greater morphological similarity to *B. deltophylla*, from which it differs in producing a cordate basal leaf blade, this with margins of narrowly triangular teeth with a long sharp mucro, and narrowly elliptic sepals that are white or white green (vs. cordate-triangular basal leaf blades with crenately lobed margins, the teeth of the margins with a mucronate apex, some lobes doubly lobed, and widely elliptic sepals that are white or pink). Furthermore, each of the three species bears a unique geographical distribution: *B. deltophylla* and the new species *B. yangii* occur in more restricted ranges, whereas *B. calthifolia* is more widespread (**Figure 8**).

Karyotypes of *B. calthifolia* and *B. deltophylla* have been previously described in a number of studies (Shang, 1985; Yang et al., 1995; Yang, 1999, 2002, Yuan and Yang, 2006). For *B. calthifolia*, two cytotypes have been identified: diploid with 2*n* = 16 and tetraploid with 2*n* = 32 (Yang, 1999). At first, the karyotype structure of this species was described by the formula 2*n* = 6m + 4m^sat^ + 2sm + 2sm^sat^ + 2t (Shang, 1985). The karyotype was later revised and determined to exhibit the formula 8m + 4sm + 2st + 2t for diploid individuals and 2*n* = 16m + 8sm + 4st + 4t for tetraploid individuals (Yang, 1999). In later studies by the same author, for several diploid populations of *B. calthifolia*, the karyotype formula 2*n* = 6m + 4m^sat^ + 2sm + 2st + 1t + 1t^sat^ was reported, similarly with Shang’s original report (Shang, 1985; Yang, 2002; Yuan and Yang, 2006). In our study, the karyotype structure in analyzed specimens matches closely with the one from Yang’s earlier study, namely, 2*n* = 4m + 4m^sat^ + 4sm + 2st + 2t (Yang, 1999). We also detected secondary constrictions on two pairs of metacentric chromosomes. It should be noted that, according to our results and those of Yang, the fifth pair of metacentric chromosomes exhibits an arm ratio close to a borderline value between metacentric and submetacentric types, which may help explain the difference between the karyotype formulas, containing either four or five pairs of metacentrics.

The karyotype structure of *B. deltophylla* described by us also differs slightly from Yang’s results (Yang et al., 1995). According to his work, this species exhibits the formula 2*n* = 10m + 4st + 2t^sat^, whereas we obtained a similar formula of 2*n* = 10m + 2sm + 2st + 2st^sat^. The specimens that we examined are characterized by the presence of a sixth pair of submetacentric chromosomes with an arm ratio of 2.58 ± 0.22. In prior work, this chromosome has an intermediate value between the sm and st types: 3.02. The eighth pair of chromosomes in our specimens displays an arm ratio close to a borderline value with the telocentric type, but we formally assigned it to the submetacentric type. The arm ratio of unequal-armed chromosomes varies more substantially among metaphase plates than that of equal-armed chromosomes, a phenomenon which may be due to uneven compaction when short arms are compared with long arms, thus potentially explaining discrepancies in findings among materials of *B. deltophylla*. The karyotype of the new species *B. yangii* is similar to the karyotype of *B. deltophylla* but shows differences from it in the position of secondary constrictions and in the morphology of fifth-pair chromosomes (see Results). Thus, chromosome sets of the three species of *Beesia* exhibit species-specific features.

### 4.2 Taxonomic treatment

The above data allowed us to distinguish a new species of *Beesia* from specimens previously identified as *B. calthifolia*.

Genus ***Beesia*** Balf.f. & W.W.Sm. 1915, Notes Roy. Bot. Gard. Edinburgh 9: 63; Liangqian and Tamura, 2001, Fl. China 6: 142; Kress et al., 2003, Contr. U.S. Natl. Herb. 45 (Checkl. trees, shrubs, herbs, climbers Myanmar): 333; Mao and Dash, 2020, Fl. Pl. India Annot. Checkl. Dicot. 1: 41.

*Type*: *Beesia cordata* Balf.f. & W.W.Sm. (= *B. calthifolia* (Maxim. ex Oliv.) Balf.f. & W.W.Sm.) (see next).

*Description: Perennial herbs*. *Rhizome* creeping or ascending vigorously. *Stem* erect, simple, subscapose, elongated after flowering with several large membranaceous sheaths at base. *Basal leaves* 2–4, simple, long petiolate, blades undivided, cordate or cordate-triangular to reniform at base, round to acuminate at apex, regularly dentate, chartaceous, petioles dilated into membranaceous sheath toward base. *Cauline leaves* present. Lower cauline leaves similar to the basal leaves, but smaller in size, upper cauline leaves 1 or 2, lanceolate. *Inflorescence* racemiform in upper part, with one to three flowers fasciculate at nodes in the lower part. *Scape* simple, with membranaceous sheath at base. *Cyme* compound, with one to three sessile fascicled flowers at several nodes. *Bracts* lanceolate or subulate. *Flowers* actinomorphic, opening flat. *Sepals* 5, petaloid, white, white-green or pink, wide or narrow elliptic, acute at apex, spreading at end of flowering stage. *Petals* absent. *Stamens* numerous, shorter, equal or longer than sepals, anthers nearly globose, lateral, filaments filiform or subfiliform. *Carpel* single. *Follicle* sessile, transversely veined, directed sideways, with distinct persistent stylodium. *Seeds* several in follicle, elliptic or ovoid, rugulose.

*Distribution*: Three species in China and Myanmar.

**Figure 9:**
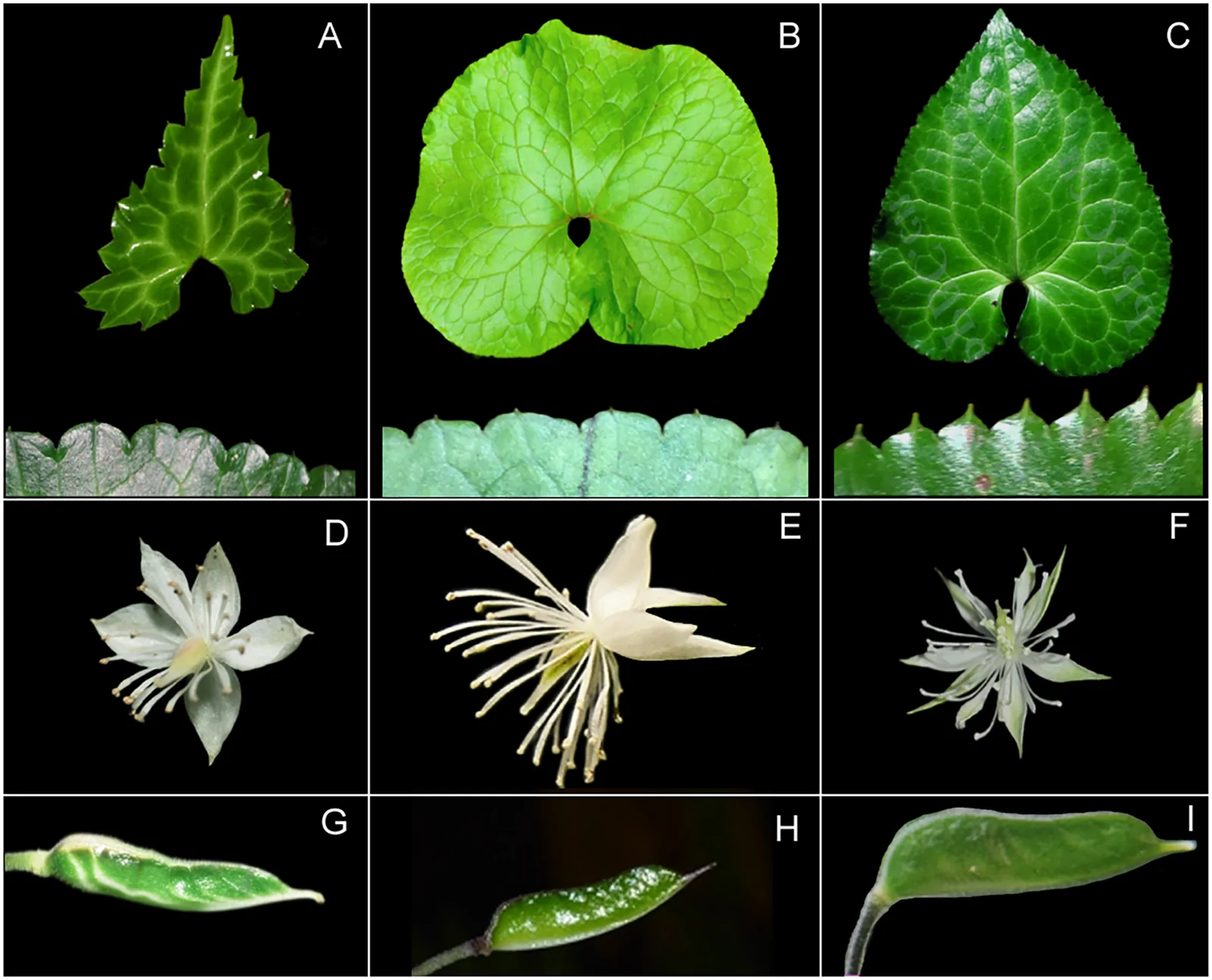
Morphological differences among species of *Beesia*: **(A, D, G)** *B*. *deltophylla*. **(B, E, H)** *B*. *calthifolia*. **(C, F, I)** *B*. *yangii.* **(A)**, **(B)**, **(C)** Leaves. **(D-F)** Flowers. **(G-I)**, (I0, Follicles.

**Figure 10:**
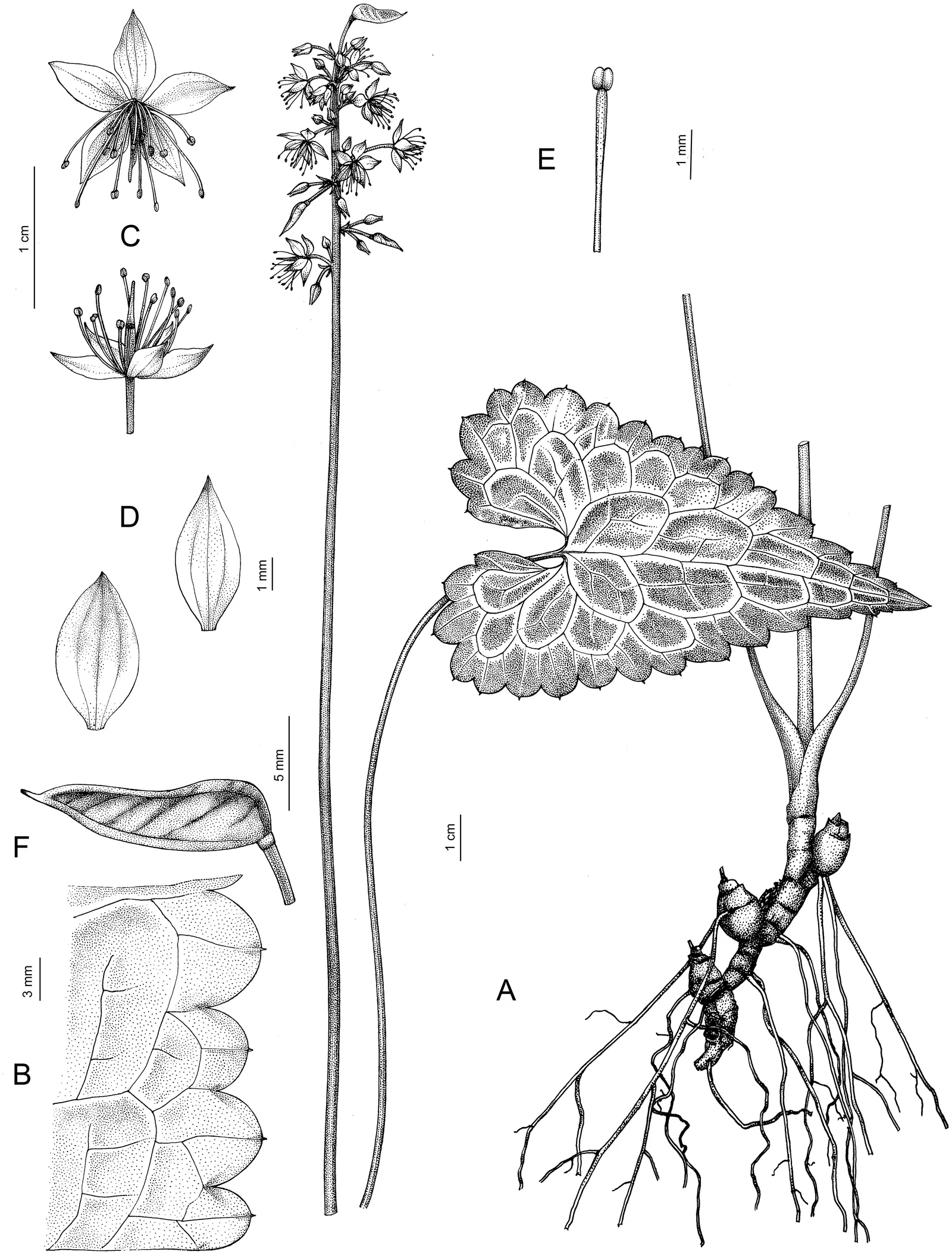
*Beesia deltophylla.* **(A)** General view. **(B)** Edge of the leaf. **(C)** Flowers. **(D)** Sepals. **(E)** Stamen. **(F)** Follicle.

1. ***Beesia deltophylla*** C.Y.Wu, 1979, Fl. Reipubl. Popularis Sin. 27: 604; Li and Tamura, 2001, Fl. China 6: 142. (**Figures 9A, D, G**, **10**).

*Type*: CHINA. Tibet, Metuo, Lage to Hanmi, 2300 m, in the broad-leaved forest on the hillside, white flowers, 3 August, 1974, *P.G. Xiao 74–3975* [in Chinese] (holotype, PE! barcode PE00028304; isotype, PE! barcode PE00440501).

– *Beesia calthifolia* auct. non (Maxim. ex Oliv.) Balf.f. & W.W.Sm.: U.L. Tiwari & A.A. Mao, 2016, Indian Forester 142(5): 508, with auth. comb. Ulbr.; A.A. Mao & S.S. Dash, 2020, Fl. Pl. India Annot. Checkl. Dicot. 1: 41.

*Description: Perennial herbs*. *Rhizome* up to ~10 × 0.3–0.7 cm, pink or purple. *Scapes* more than 14 cm at anthesis (–34 cm at fruiting) × 1–2 mm, basally subglabrous, apically spreading white pubescent with unicellular lageniform trichomes. *Basal leaves* 2 or 3; petiole 5.5–14.5 cm long, densely spreading white pubescent, dilated into membranaceous sheath toward base; leaf blade ovate-triangular, 4–9 × 2.8–5.9 cm, both surfaces glabrous, abaxially pale green, adaxially dark green with light green or white veins, glossy; base deeply cordate, margin sparsely 7–16-dentate on each side, teeth 3–8 mm wide at base, apex acuminate to long acuminate. Lower *cauline leaves* rarely present, same as basal leaves, but smaller in size, upper cauline leaves 1 or 2, lanceolate. *Inflorescence* paniculate-cymose, 5–10 сm long; bracts subulate, 1–4 mm long. *Flowers* 6–7 mm in diam.; *pedicels* 5–10 mm long, densely spreading pubescent. *Sepals* white or pink, ovate-elliptic, 4–4.5 × 2–2.5 mm, glabrous, apex acute. *Stamens* 4–6 mm long, equal to or mostly longer than the sepals; *anthers* yellowish, 0.25 mm long. *Follicle* green, sometimes turning yellowish brown, ca. 1 cm long, flat, lanceolate-linear, sparsely pubescent, with ca. 4 obliquely transverse veins; style persistent, 1–2 mm long. *Seeds* several, ca. 2 mm long, obliquely corrugate.

*Phenology:* Flowering April to June; fruiting late May to September.

*Distribution:* China, SE Xizang (Medog Xian) (**Figure 8**).

*Habitat:* Dense mixed forest, occurring on slopes; elevation: 1500–4000 m.

*Additional* sp*ecimens examined*: СHINA, Tibet, Medog County. Nyingchi, near Hanmi village, 2,227 m, 29°22′01.8″″N 95°07′05.2″E, 02 July 2024, *J. Zhang, T. Gao, Y.Y. Ling* CH2024-22-1 (NS! barcode NS0033870, PE)!; Duoxiong River, 2477 m, 29°24′10.1″N 95°05′16.3″E, 02 July 2024, *iidem* SP-7-2-1 (NS! barcode NS0033869, PE)!; Nyingchi, near Rizhalu village 2,365 m, 29°23′05.7″N 95°06′07.4″E, 02 July 2024, *iidem* SP-7-2-2 (NS! barcode NS0033868, PE)!. INDIA, Arunachal Pradesh. Lalung, Pachakshiri [2,438 m], 6 May 1938, *F. Ludlow & G. Sherriff* 3,714 (BM! barcode BM000078370); 1,829–2,134 m, 28°21′N 96°37″E, 20 July 1928, *F. Kingdon Ward* 8473 (K! barcodes K004189054 (sheet 1/2) and K004189055 (sheet 2/2)); 2,118 m, 27°34′21.6″N, 93°55′22.7″E, 04 April 2009, *A.A. Mao* 19143 (ARUN)!; 1,500 – 2,400 m, 28°46′57.1″N 95°11′33.8″E, 21 July 2010, *Pathak & Bhaumik* 72901 (CAL)!; 2,370–4,000 m, 28°46′59.9″N 95°16′51.6″ E, 11 September 2011, *Pathak* & *Gopal Krishna* 54264 (CAL)!.

*Preliminary conservation status:* Population sizes are likely decreasing with a small number of individuals, with an estimated EOO of 7,633.09 km^2^ and AOO of 44 km^2^. Thus, *B. deltophylla* was assessed as vulnerable (VU B1a+B2a,b(iii,iv)) at the global level.

**Figure 11:**
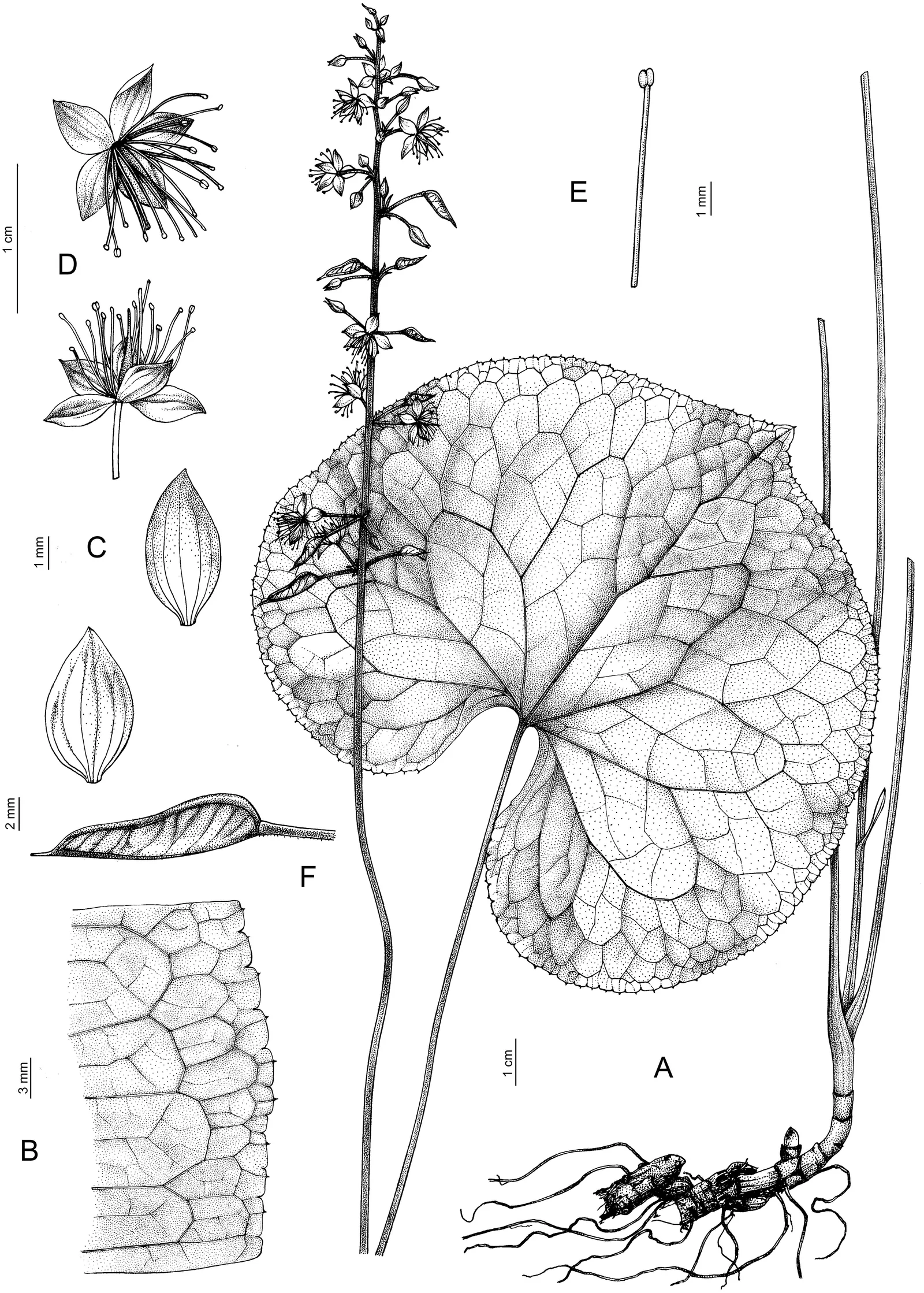
*Beesia calthifolia*. **(A)** General view. **(B)** Edge of the leaf. **(C)** Flowers. **(D)** Sepals. **(E)** Stamen. **(F)** Follicle.

2. ***Beesia calthifolia*** (Maxim. ex Oliv.) Balf.f. & W.W.Sm. 1924, Notes Roy. Bot. Gard. Edinburgh, 14: 110; Ulbr. 1929, Notizbl. Bot. Gart. Berlin-Dahlem 10: 872, isonym; Li and Tamura, 2001, Fl. China 6: 142; Kress et al., 2003, Contr. U.S. Natl. Herb. 45 (Checkl. trees, shrubs, herbs, climbers Myanmar): 333. ≡ *Cimicifuga calthifolia* Maxim. ex Oliv. 1888, Hooker’s Icon. Pl. 18: pl. 1746 (as *calthaefolia*). (**Figures 9B, E, H**, **11**).

*Type:* CHINA. Szechwan, Mount Omei, 4500 ft, *E. Faber* 624 (lectotype, K! barcode K004189049, selected here by Erst, Borosova and Tatanov). *Syntypes: ibidem*, 6,000 ft, *idem* 625 (var. *minor*) (K! barcode K004189053, LE! barcode LE01013623, NY! barcode NY00233186 (left-hand fragments on the sheet)); *ibidem*, summit in ravine, *idem* 626 (var. *minor*) (K! barcode K004189052, NY! barcode NY00233186 (right-hand plant on the sheet)); China borealis, prov. Kansu orientali, fl. Dshombunou, prope montem Tschagola, 10 July 1885, *G.N. Potanin* (LE! barcode LE01013622); China borealis, prov. Kansu orientali, in monte Itschu-schan, 15 July 1885, *idem* (LE! barcodes LE01013624 and LE01320481); Kansu orientali, 1885, recd. 4 Sept. 1890, *idem* (K! barcode K004189051, P! barcode P00200370).

= *Beesia cordata* Balf.f. & W.W.Sm. 1915, Notes Roy. Bot. Gard. Edinburgh 9: 63. – *Lectotype:* West China. Yunnan, open moist pasture on the margins of thickets on the Kari Pass, Mekong-Yangtze divide, Lat. 27°40″N, Alt. 9,000–10,000 ft, Aug. 1914, *G. Forrest* 12955 (E! barcode E01150776, selected here by Erst, Borosova and Tatanov). *Syntype:* Northern Burma [Myanmar], Feng-shui-ling Camp near the Burma-China frontier, among undergrowth of rain forest in deep shade, wet clayey soil, 9,000 ft, fls. white, 9 June 1914, *F. Kingdon Ward* 1660 (E! barcodes E00078798 (sheet 1/2) and E00078799 (sheet 2/2)).

= *Beesia elongata* Hand.-Mazz. 1922, Anz. Akad. Wiss. Wien, Math.-Naturwiss. Kl. 59: 245. – *Lectotype*: China, Yünnan, prope fines Tibeto-Birmanicas inter fluvios Lu-djiang (Salween) et Djiou-djiang (Irrawadi or. Sup.), in jugi Tschiangschel, 27°52″N, 5 July 1916, *H.F. Handel-Mazzetti* 9156 (WU! barcode WU0033932, selected here by Erst, Borosova and Tatanov; isolectotype, E! barcode E00064955).

– *Cimicifuga calthifolia* var. *minor* Maxim. ex Oliv. 1888, Hooker’s Icon. Pl. 18: pl. 1746, nom. inval., without description.

*Description: Perennial herbs*. *Rhizome* up to 10 × 0.3–0.5 cm, yellow or light brown. *Scapes* more than 14 cm at anthesis (– 58 cm at fruiting) × 1–2 mm, basally glabrous, apically densely pubescent with unicellular lageniform trichomes. *Basal leaves* 2–4; petiole (5.5–)10–26 cm × 0.5–1.5 mm, glabrous, dilated into membranaceous sheath toward base; leaf blade reniform, orbicular-ovate, (1.5–)4.5–9.5 × (1.8–)5.5–16 cm, both surfaces glabrous, rarely pubescent abaxially on veins, light green with inconspicuous veins, dull, base deeply cordate, margin dense with 40–50 teeth on each side, teeth widely crenate with long mucro at apex, sometimes off-center, apex rounded or shortly acuminate. Lower *cauline leaves* present, same as basal leaves, but smaller in size, upper cauline leaves 1 or 2, lanceolate. *Inflorescence* cymose, 5.5–9.5 × 1.5–2.5 cm; bracts usually subulate, sometimes lanceolate, rarely spatulate, 1–1.5 mm long, glabrous. *Flowers* 0.8–1.1 cm in diam.; *pedicels* 5–10 mm long, densely spreading pubescent. *Sepals* white or pinkish, narrowly ovate or elliptic, 4–6 × 1.5–2 mm, glabrous, apex acute or rounded. *Stamens* 3–3.1 mm long, shorter than sepals; anthers 0.3 mm long. *Follicle* green, 1.1–1.7 cm long, flat, lanceolate-linear, middle part curved, lower part 3–4 mm wide, sparsely pubescent near base, otherwise glabrous, with ca. 8 obliquely transverse veins; style persistent, 1–2 mm. *Seeds* several, ca. 2.5 mm long, obliquely corrugate.

*Phenology:* Flowering March to July; fruiting late April to September.

*Distribution:* China (S. Gansu, N. Guangxi, Guizhou, W. Hubei, W. Hunan, S. Shanxi, Sichuan, N.W. Yunnan); Myanmar (North) (**Figure 8**).

*Habitat:* Rainforest and open woodland, occurring on wooded rocky slopes, on river banks, moist slopes along streams, in deep shade amongst undergrowth; elevation: 1,400–4,425 m.

*Additional* sp*ecimens examined.* CHINA. [Chongqing] Cheng-kou, 1,450 m, 12 April, 1958, *T.L. Tai* 102623 (E! barcode E00064958); *ibidem*, 1,430 m, 26 May 1958, *idem* 100630 (E! barcode E00064959). Guizhou. Chire-kang-Viheon, May 1908, *Cavalerie* 2949 (K! barcode K004189038, P! barcodes P00200356 (sheet 1/3), P00200357 (sheet 2/3) and P00200358 (sheet 3/3)). Hupeh [Hubei]. 1885–1888, *A. Henry* 5957 (BM! barcode BM015177131, E! barcode E01150770, LE! barcode LE01320483, P! barcode P00840973, US! barcode US03558171); *ibidem*, 1885–1888, *idem* 5790 (BM! barcode BM015177132, E! barcode E00064956, US! barcode US03558172); *ibidem*, 1885–1888, *idem* 5790B (BM! barcode BM000078365, P! barcode P00840975, US! barcode US801343); Patung, May 1888, *idem* 4669 (K! barcode K004189050, LE! barcode LE01320482); Kuei, recd. March 1889, *idem* 5790A (BM! barcode BM000078364, K! barcode K004189045, US! barcode US03558173); Ichang, 26 Dec. 1889, *idem* 5432 (P! barcode P00200369); July 1900, *E.H*. *Wilson* 1292 (E! barcode E01150768, K! barcode K004189041, LE! barcode LE01320484, NY! barcode NY04991245, P! barcode P00200363); *ibidem*, July 1907, *idem* 2322 (K! barcode K004189044). Kansu [Gansu], Yanchang, Gongjiagou, 2,450 m, 15 July 1989, *Y.M. Yuan* 1052 (BM! barcode BM015177133, E! barcode E01150778). Shanxi. Wutaishan, above Huadianba, N end of Cangshan, 3,300 m, 20 May 1981, *Sino-British expedition to Cangshan* 918 (E! barcode E01150777, K! barcode K004189040). [Sichuan] Omei [3,353 m], July 1887, *E. Faber* 668 (E! barcode E01150780); recd. March 1889, *A. Henry* 7220 (K! barcode K004189045); Tachienlu [Kangding], [2,743–4,115 m] purchased, Dec. 1890, *A.E. Pratt* 823 (BM! barcode BM000078371, E! barcode E01150769, K! barcode K004189047, LE! barcode LE01320488, P! barcodes P00200371 (sheet 1/4), P00200372 (sheet 2/4), P00200373 (sheet 3/4) and P00200374 (sheet 4/4)); Mt Omei, June 1904, *Wilson* (P! barcode P00200375); *ibidem*, *idem* 4712 (K! barcode K004189043); 3,800 m, 27 May 1914, *C. Schneider* 1406 (E! barcode E01041927); Dongrergo, 3,900 m, 23 July 1923, *H. Smith* 3773 (E! barcode E01150782, K! barcode K004189059); Kulu, E of Muli Gomba, 3650–4425 m, June 1928, *F. Rock* 16473 (E! barcode E00064957, NY! barcode NY04419612, US! barcode US03558165); Sungpan hsien, 10 Aug. 1928, *W.P. Fang* 4142 (E! barcode E01150771, K! barcode K004189035, US! barcode US03558163); Omei-shan, 18 Aug. 1928, *idem 3161* (E! barcode E01150774); *ibidem*, 14 June 1930, *idem* 4731 (US! barcode US03558164); *ibidem*, 13 July 1930, *idem* 7468 (K! barcode K004189036); *ibidem*, 15 July 1930, *idem* 7606 (K! barcode K004189037); Mt Omei, 2,000 m, 15 July 1931, *F.T. Wang* 23333 (LE! barcode LE01300485); Tahsiangling, 2,400 m, 26 June 1934, *H. Smith* 10181 (BM! barcode BM015177135); Sikang, Kangting (Tachienly), 3,300 m, 15 July 1934, *idem* 10468 (BM! barcode BM015177136, E! barcode E00064960, NY! barcode NY04419611); Tien-chuan-hsien, 2,400 m, 25 May 1936, *K.L. Chu* 2653 (BM! barcode BM015177139); *ibidem*, 3,200 m, 13 June 1936, *idem* 2773 (BM! barcode BM000078363, E! barcode E01150773); Pao-hsing-hsien, 25 July 1936, *idem* 3292 (BM! barcode BM015177137, E! barcode E01150783); *ibidem*, 3,000 m, 11 Aug. 1936, *idem* 3527 (BM! barcode BM015177134); *ibidem*, 3,000 m, 11 Aug. 1936, *idem* 3530 (BM! barcode BM015177138, E! barcode E01150779); Kangting, 1963, *Kuan & Wang* 1303 (K! barcode K004189039); Luding Xian, Moxi, Gongga Shan, E side, 3,000–3,100 m, *D.E. Boufford* et al., 27382 (E! barcode E00647472, NY! barcode NY04419615, P! barcode P02548475); Dujianyan, from Mashanping to Qinglongzui along the Longxi River, 1,400 m, 11 Sept. 1988, *D.E. Boufford* et al., 24815 (NY! barcode NY04419613); Mianning Xian, Lamagetou Nature reserve, Yele Xiang 2,790–3,440 m, 10 July 2005, *D.E. Boufford* et al. 32782 (P! barcode P02548474); Tchen-keou-tin, Su-tchuen oriental., 1,400 m, *R.P. Farges* 64 (K! barcode K004189042, LE! barcode LE01320486, NY! barcode NY04419610, P! barcodes P00200366 (sheet 1/3), P00200367 (sheet 2/3) and P02527942 (sheet 3/3)); Wenchuan, Ngawa Tibetan and Qiang Autonomous Prefecture, Wolong, 2,803 m, 06 July 2024, *A.S. Erst*, *T.V. Erst*, *D.A. Krivenko*, *H.W. Peng* CH2024-13 (IRK barcode IRK00049786, NS barcodes NS0003871 and NS0003872, PE). Yunnan. San Chan, May 1906, *F. Ducloux* 133 (E! barcode E01150781); *ibidem*, May 1906, *idem* 4722 (P! barcodes P00200364 (sheet 1/2) and P00200365 (sheet 2/2)); *ibidem*, 12 June 1907, *idem* 5188 (P! barcodes P00200359 (sheet 1/2) and P00200360 (sheet 2/2)); Dschungdien (Chuntien) et viscum Djitsung, 4,000–4,100 m, 24 Aug. 1915, *H.F. Handel-Mazzetti* 7759 (WU! barcodes WU033929 (sheet 1/2) and WU033930 (sheet 2/2)); Landsang-djiang (Mekong) et Lu-djiang (Salween), 3,200–3,500 m, 16 June 1916, *idem* 8917 (WU! barcode WU033931); [3,353–3,658 m] July 1921, *G. Forrest* 19707 (CAL! barcode CAL0000041281, E! barcode E01150775, K! barcode K004189058, P! barcode P00200361); [3,353–3,658 m] Aug. 1921, *idem* 19907 (US! barcode US03558169); Ku-long-tchiang, 800 m, 10 Aug. 1921, *R.P. Maire* (P! barcode P00200362); Mekong-Salwin divide [3,353 m], Sept. 1921, *G. Forrest* 20390 (E! barcode E01150772, K! barcode K004189057, US! barcode US03558168); Mountains above Tseku and Tsehchung, Mekong-Salween watershed, 3,048–3,353 m, 1923, *J.F. Rock* 8729 (US! barcode US03558167, NY! barcode NY04419616); Mount Lauchunshan, SW of the Yangtze bend at Shiku, June 1923, *idem* 9568 (US! barcode US03558166); Diqing, Zhongdian, below Tian Shu lake, above Xiaozhongdian, 3800 m, 13 June 1993, *B. Alden* et al., 1231 (E! barcode E011507670); Zhongdian, Tianchi lake, 11 June 1994, *Ace* 143 (K! barcode K004189048); Shangrila Xian, near Tianchi lake, 3,700 m, 24 July 2007, *Tibet-Mac Arthur* 1216 (US! barcode US00972018); Muli Xian, near Chang-Hai-Zi lake, 3,674 m, 4 Aug. 2007, *idem* 1779 (US! barcode US00994674); Shangrila Co., Xiao Zhongdian, on the road to Tianchi lake, 3,834 m, 16 June 2009, *idem* 2357 (US! barcode US00863424); *ibidem*, 16 June 2009, *idem* 2343 (US! barcode US00863410); *ibidem*, 16 June 2009, *idem* 2309 (US! barcode US00863295). MYANMAR. Northern Burma, below Feng-shui-ling Camp, near the Burma-China frontier [2743 m], June 1914. F*. Kingdon Ward* 1660 (E! barcode E00078799); Upper Burma, divide between Feng-shui-ling and Lai Kam valleys, 9 Sept. 1919, *R.J. Farrer* 27382 (E! barcode E00078800). See аlso type specimens above.

*Preliminary conservation status: Beesia calthifolia* is a widespread species in China. The estimated EOO is 922,109.8 km^2^ and the AOO is 124 km^2^. Thus, *B. calthifolia* was assessed as least concern at the global level.

**Figure 12:**
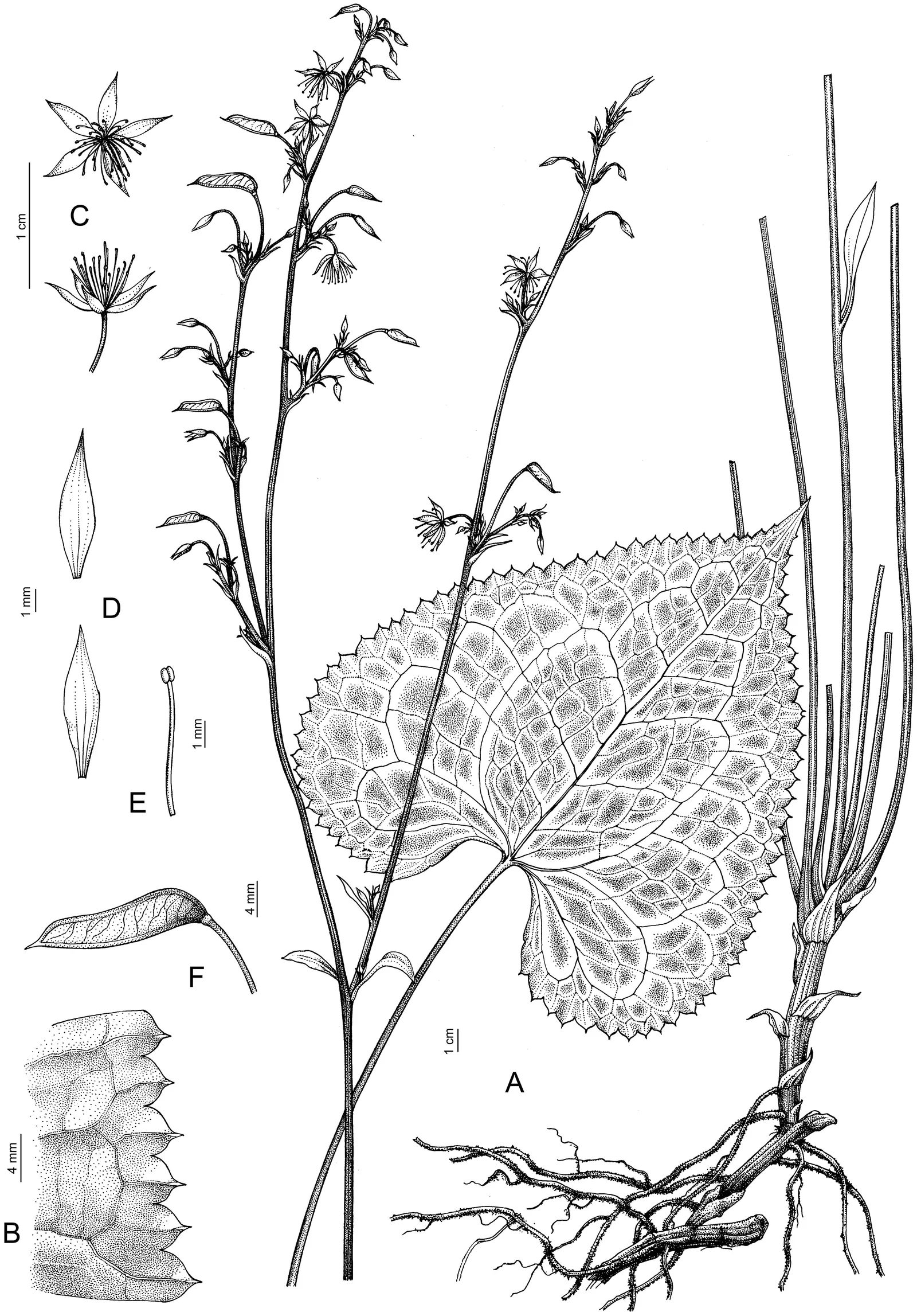
*Beesia yangii*. **(A)** General view. **(B)** Edge of the leaf. **(C)** Flowers. **(D)** Sepals. **(E)** Stamen. **(F)** Follicle.

3. *Beesia yangii* Erst & K.L. Xiang, sp. nov. (**Figures 9C, F, I**, **12**). urn:lsid:ipni.

org:names: 77371025-1

*Type:* CHINA. Sichuan Province, Dujiangyan Mount, Quingcheng, 970 m, 30°54′18.5″N 103°33′13.6″E, 18 April 2024, *Erst A.S.*, *Erst T.V.*, *Zhang J.* CH2024-5 (holotype, NS barcode NS0000490; isotypes, IRK barcode IRK00058024, LE barcode LE01323001, NS barcodes NS0000491 and NS0000492). *Paratypes:* CHINA. Sichuan Province, Pengzhou, Tangba village, 1186 m, 31°11′37.1″N 103°56′29.1″E, 27 May 2025, *Erst A.S., Erst T.V., Zhou X.P., Zhu D.L.* CH2025-22 (IRK barcode IRK00059177, LE barcode LE01323217, NS barcode NS0000700).

*Description: Perennial herbs. Rhizome* up to 10 × 0.4–1.1 cm, pink or purple. *Scapes* more than 40(–75) cm at fruiting × 1–2 mm, basally glabrous, apically densely pubescent. *Basal leaves* 2–4; petiole (5.5)10–26 cm × 1–2 mm, glabrous, dilated into membranaceous sheath toward base; leaf blade cordate, (1.9–)4.5–13.5 × (3–)5.5–12 cm, both surfaces glabrous, abaxially pale green, adaxially dark-green with light-green or white veins, glossy, base cordate, margin dense with 13–50 teeth on each side, teeth narrowly triangular with long sharp mucro, apex acuminate to acute. Lower *cauline leaves* same as basal leaves but smaller in size, upper cauline leaves 1 or 2, lanceolate. *Inflorescence* cymose, 5.5–9.5 × 1.5–2.5 cm; bracts usually subulate, sometimes lanceolate, rarely spatulate, 1–1.5 mm long, glabrous. *Pedicels* 5–10 mm long, densely spreading pubescent. *Sepals* white or white-green, narrowly elliptic, 5–9 × 2–4 mm, glabrous, apex acute, or rounded. *Stamens* 0.4–0.8 mm long, equal to or mostly shorter than sepals; anthers 0.3 mm long. *Follicle* (0.7–)1.1–1.7(–1.8) cm long, flat, lanceolate-linear, middle part curved, lower part 3–4 mm wide, mostly glabrous, sparsely pubescent near base, with ca. 8 obliquely transverse veins; style persistent, 1–2 mm. Seeds several, ca. 2.5 mm long, obliquely corrugate.

*Affinity:* The new species is sister to *B. calthifolia* and *B. deltophylla*, according to the results of molecular phylogenetic analysis (**Figure 4**). *Beesia yangii* is morphologically similar to *B. calthifolia* and *B. deltophylla* (**Figure 9**) in having simple long petiolate basal undivided leaves with cordate or cordate-triangular to reniform blades, crenulate-mucronate leaf margins, paniculate-cymose inflorescences, actinomorphic flowers with sepals, petals lacking, one follicle per flower, and thickened underground creeping rhizomes that produce new aerial shoots. The differences among the three species are presented in **Table 1**. The new species differs from related species by bearing a cordate basal leaf blade, this with reliably toothed margins, the teeth narrowly triangular with a reliably long sharp mucro, and narrow elliptic, white, or white-green sepals. The sepals are shorter than or equal to the stamens. Additionally, all three species have different distribution patterns (**Figure 8**).

*Phenology:* Flowering March to early April; fruiting late April to May.

*Distribution*: Endemic to Dujiangyan Mount, Sichuan Province, China (**Figure 8**).

*Habitat:* Forests, occurring on slopes, elevation: 900–1,700 m.

*Etymology:* The specific epithet ‘yangii’ of the new species is given in honor of the famous researcher of representatives of the Ranunculaceae family in China: Qin-Er Yang.

*Preliminary conservation status:* The newly described species is so far known only from two isolated locations; the estimated AOO is 8 km^2^. Thus, *B. yangii* was assessed as critically endangered (CR B2 a, b (ii, iii, iv, v)) at the global level.

### 4.3 Identification key to species of *Beesia*

1. Basal leaf blade reniform, light green, with inconspicuous veins, a dull surface, apex rounded or acuminate, edge of basal leaves toothed, teeth widely crenate with long mucro at apex, sometimes off-center; rhizome yellow or light brown; sepals shorter than stamens in open flowers …………………………………………….*B. calthifolia*

+ Basal leaf blade cordate or cordate-triangular, dark green, with lighter green veins, glossy surface, apex acuminate, edge of basal leaves toothed or crenately lobed, teeth narrowly triangular with long sharp mucro or each lobe with mucronate apex, some lobes doubly lobed; rhizome pink or purple; sepals longer than or equal to stamens in open flowers …………….……………………2

2. Basal leaf blade cordate-triangular, edge of basal leaves crenately lobed, teeth in each lobe with mucronate apex, some lobes doubly lobed; sepals wide elliptic, white or pink…..…. *B. deltophylla*

+ Basal leaf blade cordate, edge of basal leaves toothed, teeth narrowly triangular with long sharp mucro; sepals narrowly elliptic, white or white green ………………………….…………… *B. yangii*

## 5 Conclusion

The integrative taxonomic approach applied in this study using different types of data, including numerous molecular markers together with numerical and qualitative morphological metadata, identified three taxa, including the new cryptic species *B. yangii*. These data demonstrate that the new taxon is distinct according to comprehensive data showing that it is morphologically and cytogenetically unique, as well as monophyletic. Broader application of integrative approaches may continue to yield important new discoveries in both systematic biology and conservation of biodiversity.

## Data Availability

The datasets presented in this study can be found in online repositories. The names of the repository/repositories and accession number(s) can be found in the article/**Supplementary Material**.
