# Correction: A revision of the genus *Beesia* (Ranunculaceae) as informed through integrative taxonomy, with description of a new species from Sichuan (China)

**DOI:** 10.3389/fpls.2026.1787443

**Published:** 2026-02-12

**Authors:** Andrey S. Erst, Elizaveta Yu. Mitrenina, Denis A. Krivenko, Tatyana V. Erst, Yulia V. Cheldysheva, Igor V. Gorbenko, Renata Borosova, Lian Lian, Yuan-Yuan Ling, Huan-Wen Peng, Jun Zhang, Shukherdorj Baasanmunkh, Hyeok Jae Choi, Ivan V. Tatanov, Alexander A. Kuznetsov, Mathew T. Sharples, Kun-Li Xiang

**Affiliations:** 1 Laboratory of Herbarium, Central Siberian Botanical Garden of the Siberian Branch of the Russian Academy of Sciences, Novosibirsk, Russia; 2 Department of Genetics and Cell Biology, Biological Institute, National Research Tomsk State University, Tomsk, Russia; 3 Department of Biodiversity and Biological Resources, Siberian Institute of Plant Physiology and Biochemistry of the Siberian Branch of the Russian Academy of Sciences, Irkutsk, Russia; 4 Laboratory of Molecular Phytopathology, Institute of Cytology and Genetics of the Siberian Branch of the Russian Academy of Sciences, Novosibirsk, Russia; 5 Laboratory of Biological Control of Phytophages and Phytopathogens, Siberian Federal Scientific Centre of Agrobiotechnologies of the Russian Academy of Sciences, Krasnoobsk, Russia; 6 Laboratory of Plant Genetic Engineering, Siberian Institute of Plant Physiology and Biochemistry of the Siberian Branch of the Russian Academy of Sciences, Irkutsk, Russia; 7 Herbarium, Royal Botanic Gardens, Kew, London, United Kingdom; 8 State Key Laboratory of Plant Diversity and Specialty Crops, Institute of Botany, Chinese Academy of Sciences, Beijing, China; 9 China National Botanical Garden, Beijing, China; 10 University of Chinese Academy of Sciences, Beijing, China; 11 Department of Biology and Chemistry, Changwon National University, Changwon, Republic of Korea; 12 Department of Herbarium of Higher Plants, Komarov Botanical Institute of the Russian Academy of Sciences, St. Petersburg, Russia; 13 Laboratory of the Herbarium, National Research Tomsk State University, Tomsk, Russia; 14 Department of Biological and Earth Sciences, Arkansas Tech University, Russellville, AR, United States

**Keywords:** *Beesia*, China, genome, karyotype, integrative taxonomy, new species, phylogeny, Ranunculaceae

There was a mistake in **Figure 8** as published. Some distribution points on the previous figure were indicated. The corrected **Figure 8** appears below.

**Figure 8:**
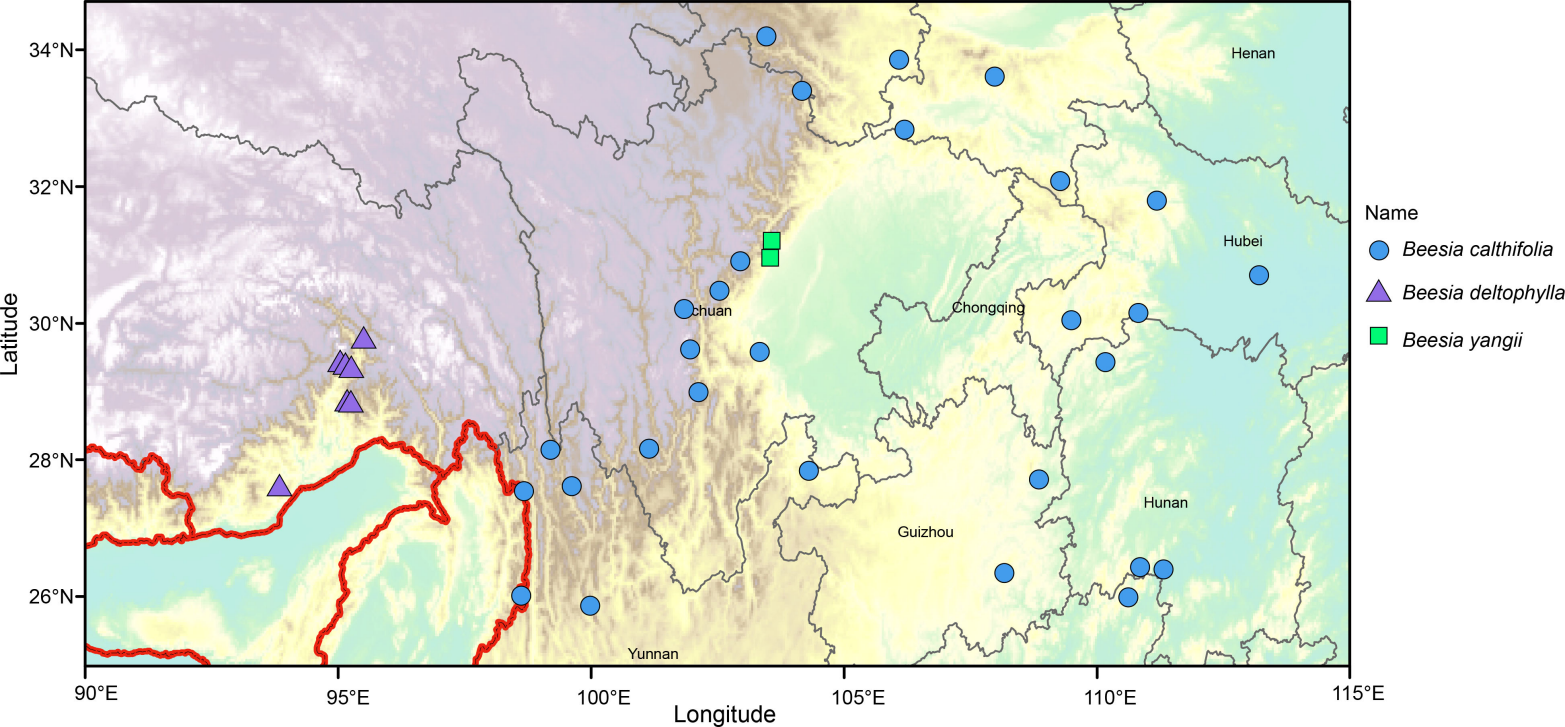
Distribution map of known localities of occurrence for species of Beesia.

“A correction has been made to section 4 Discussion, 4.2 Taxonomic treatment, Paragraph 5. The sentence originally stated “*Distribution*: Three species in China, India and Myanmar.” The corrected sentence states “*Distribution*: Three species in China and Myanmar.”

A correction has been made to section 4 Discussion, 4.2 Taxonomic treatment, Paragraph 11. The sentence originally stated “*Distribution*: China, SE Xizang (Mêdog Xian); India, Arunachal Pradesh (upper Siang) **Figure 8**).” The corrected sentence states “*Distribution*: China, SE Xizang (Medog Xian) (**Figure 8**).”

A correction has been made to section 4 Discussion, 4.2 Taxonomic treatment, Paragraph 13. The original paragraph stated “*Additional specimens examined*: СHINA, Tibet, Mêdog County. Nyingchi, near Hanmi village, 2,227 m, 29°22′01.8″″N 95°07′05.2″E, 02 July 2024, *J. Zhang, T. Gao, Y.Y. Ling* CH2024-22-1 (NS! barcode NS0033870, PE)!; Duoxiong River, 2477 m, 29°24′10.1″N 95°05′16.3″E, 02 July 2024, *iidem* SP-7-2-1 (NS! barcode NS0033869, PE)!; Nyingchi, near Rizhalu village 2,365 m, 29°23′05.7″N 95°06′07.4″E, 02 July 2024, *iidem* SP-7-2-2 (NS! barcode NS0033868, PE)!. INDIA, Arunachal Pradesh. Lalung, Pachakshiri [2,438 m], 6 May 1938, *F. Ludlow & G. Sherriff* 3,714 (BM! barcode BM000078370); Delei valley [1,829–2,134 m], 28°21′N 96°37″E, 20 July 1928, *F. Kingdon Ward* 8473 (K! barcodes K004189054 (sheet 1/2) and K004189055 (sheet 2/2)); Lower Subansiri district, Pange-Talle valley road, 2,118 m [27°34′21.6″N 93°55′22.7″E], 04 April 2009, *A.A. Mao* 19143 (ARUN)!; Siang, Singa to Sitoma, 1,500–2,400 m [28°46′57.1″N 95°11′33.8″E], 21 July 2010, *Pathak & Bhaumik* 72901 (CAL)!; Upper Siang, Sitoma to Pemashree, 2,370–4,000 m [28°46′59.9″N 95°16′51.6″E], 11 September 2011, *Pathak & Gopal Krishna* 54264 (CAL)!.”

The corrected sentence states “*Additional specimens examined*: CHINA, Tibet, Medog County. Nyingchi, near Hanmi village, 2,227 m, 29°22′01.8″″N 95°07′05.2″ E, 02 July 2024, *J. Zhang, T. Gao, Y.Y. Ling* CH2024-22-1 (NS! barcode NS0033870, PE)!; Duoxiong River, 2477 m, 29°24′10.1″N 95°05′16.3″E, 02 July 2024, *iidem* SP-7-2-1 (NS! Barcode NS0033869, PE)!; Nyingchi, near Rizhalu village 2,365 m, 29°23′ 05.7″N 95°06′07.4″E, 02 July 2024, *iidem* SP-7-2-2 (NS! Barcode NS0033868, PE)!; 2,438 m, 6 May 1938, *F. Ludlow & G. Sherriff* 3,714 (BM! Barcode BM000078370); 1,829–2,134 m, 28°21′N 96°37″E, 20 July 1928, *F. Kingdon Ward* 8473 (K! barcodes K004189054 (sheet 1/2) and K004189055 (sheet 2/2)); 2,118 m, 27°34′21.6″N, 93°55′22.7″E, 04 April 2009, *A.A. Mao* 19143 (ARUN)!; 1,500 – 2,400 m, 28°46′57.1″N 95°11′33.8″E, 21 July 2010, *Pathak & Bhaumik* 72901 (CAL)!; 2,370–4,000 m, 28°46′59.9″N 95°16′51.6″E, 11 September 2011, *Pathak & Gopal Krishna* 54264 (CAL)!.”

In the acknowledgements, the sentence “we thank Manas Bhaumik (Botanical Survey of India – Industrial Section Indian Museum, Kolkata) and Umeshkumar Tiwari (Botanical Survey of India – Arunachal Pradesh Regional Centre, Itanagar) for clarifying the location of Beesia deltophylla in India” has been corrected to “we thank Manas Bhaumik (Botanical Survey of India – Industrial Section Indian Museum, Kolkata) and Umeshkumar Tiwari (Botanical Survey of India – Arunachal Pradesh Regional Centre, Itanagar) for providing some information on the *Beesia deltophylla* specimens”.

The original version of this article has been updated.

