# Correction: A revision of the genus *Beesia* (Ranunculaceae) as informed through integrative taxonomy, with description of a new species from Sichuan (China)

**DOI:** 10.3389/fpls.2026.1991081

**Published:** 2026-10-07

**Authors:** Andrey S. Erst, Elizaveta Yu. Mitrenina, Denis A. Krivenko, Tatyana V. Erst, Yulia V. Cheldysheva, Igor V. Gorbenko, Renata Borosova, Yuan-Yuan Ling, Huan-Wen Peng, Jun Zhang, Shukherdorj Baasanmunkh, Hyeok Jae Choi, Ivan V. Tatanov, Alexander A. Kuznetsov, Mathew T. Sharples, Kun-Li Xiang

**Affiliations:** 1 Laboratory of Herbarium, Central Siberian Botanical Garden of the Siberian Branch of the Russian Academy of Sciences, Novosibirsk, Russia; 2 Department of Genetics and Cell Biology, Biological Institute, National Research Tomsk State University, Tomsk, Russia; 3 Department of Biodiversity and Biological Resources, Siberian Institute of Plant Physiology and Biochemistry of the Siberian Branch of the Russian Academy of Sciences, Irkutsk, Russia; 4 Laboratory of Molecular Phytopathology, Institute of Cytology and Genetics of the Siberian Branch of the Russian Academy of Sciences, Novosibirsk, Russia; 5 Laboratory of Biological Control of Phytophages and Phytopathogens, Siberian Federal Scientific Centre of Agrobiotechnologies of the Russian Academy of Sciences, Krasnoobsk, Russia; 6 Laboratory of Plant Genetic Engineering, Siberian Institute of Plant Physiology and Biochemistry of the Siberian Branch of the Russian Academy of Sciences, Irkutsk, Russia; 7 Herbarium, Royal Botanic Gardens, Kew, London, United Kingdom; 8 State Key Laboratory of Plant Diversity and Specialty Crops, Institute of Botany, Chinese Academy of Sciences, Beijing, China; 9 China National Botanical Garden, Beijing, China; 10 University of Chinese Academy of Sciences, Beijing, China; 11 Department of Biology and Chemistry, Changwon National University, Changwon, Republic of Korea; 12 Department of Herbarium of Higher Plants, Komarov Botanical Institute of the Russian Academy of Sciences, St. Petersburg, Russia; 13 Laboratory of the Herbarium, National Research Tomsk State University, Tomsk, Russia; 14 Department of Biological and Earth Sciences, Arkansas Tech University, Russellville, AR, United States

**Keywords:** *Beesia*, China, genome, karyotype, integrative taxonomy, new species, phylogeny, Ranunculaceae

An incorrect number was provided for the National Key Research and Development Program of China, as “2024YFF1701404”. The correct number is “2023YFF0805800”.

The original version of this article has been updated.

