# Supplementary material for: A revision of the genus Beesia (Ranunculaceae) as informed through integrative taxonomy, with description of a new species from Sichuan (China)
Source: Front Plant Sci. 2026 Jan 5;16:1699952. doi: 10.3389/fpls.2025.1699952 (PMC12813076; doi:10.3389/fpls.2025.1699952)
Supplement: Supplementary file 1 [file Table1.docx]

**Supplementary materials**

**Table S1. The list of *Beesia* specimens used in morphological (M), phylogenetical (P), and cytogenetic (C) analyses**

| **№** | **Taxon** | **Collector number** | **Locality** | **Coordinates, elevation** | **Сollector(s), date** | **Type of analysis** |
| --- | --- | --- | --- | --- | --- | --- |
| 1 | *B. calthifolia* | CH2024-13 | China, Sichuan Province, Wenchuan County, Ngawa Tibetan and Qiang Autonomous Prefecture, Wolong | 30°51'21.7"N, 102°58'23.5"E | A. Erst, T. Erst, D. Krivenko, H.W. Peng, 06.07.2024 (IRK, NS, PE) | M, P, C |
| 2 | *B. calthifolia* | BC2 | China, Yunnan Province, Yiliang, Zhaotong | 27.83°N, 104.29°E | ZP-135, Z. Zhou, J.C. Peng (KUN) | P |
| 3 | *B. calthifolia* | BC3 | China, Hunan Province, Shimen, Changde | 30.11°N, 110.8°E | ZDG-shimen, D.G. Zhang (planted in KIB) | P |
| 4 | *B. calthifolia* | BC4 | China, Sichuan Province, Dongla Mountain, Baoxing | 30.43°N, 102.56°E | Niu-Baoxing-014, Y. Niu (KUN) | P |
| 5 | *B. calthifolia* | BC5 | China, Sichuan Province, Emeishan, Leshan | 29.56°N, 103.34°E | ZYZ-emei, Y.Z. Zhang (photo seen) | P |
| 6 | *B. calthifolia* | BC6 | China, Yunnan Province, Lushui, Nujiang | 25.99°N, 98.66°E | PSL-002, J.C. Peng, L. Sun (planted in KIB) | P |
| 7 | *B. calthifolia* | BC7 | China, Sichuan Province, Mianning, Liangshan | 28.96°N, 102.16°E | ZP-53, Z.Zhou, J.C. Peng (KUN) | P |
| 8 | *B. calthifolia* | BC8 | China, Sichuan Province, Luding, Garze | 29.58°N, 102°E | ZP-99, Z.Zhou, J.C. Peng (KUN) | P |
| 9 | *B. calthifolia* | BC9 | China, Yunnan Province, Xizhouzhen, Dali | 25.87°N, 100°E | PSL-001, J.C.Peng, L. Sun (planted in KIB) | P |
| 10 | *B. calthifolia* | BC10 | China, Sichuan province, Kangding, Garze | 30.17°N, 101.87°E | ZP-92, Z. Zhou, J. C. Peng (KUN) | P |
| 11 | *B. calthifolia* | BC11 | China, Sichuan Province, Muli, Liangshan | 28.14°N, 101.16°E | ZP-22, Z. Zhou, J.C. Peng (KUN) | P |
| 12 | *B. calthifolia* | BC12 | China, Yunnan Province, Bi Gu Tianchi, Shangri-La, Deqen | 27.63°N, 99.64°E | ZQP-295, Z. Zhou et al. (KUN) | P |
| 1 | *B. yangii* sp. nov. | CH2024-5 | China, Sichuan Province, Dujiangyan Mount, Quingcheng | 30°54'18.5"N, 103°33'13.6"E | A. Erst, T. Erst, J. Zhang, 04.05.2024 (NS, PE) | M, P, C |
| 2 | *B. yangii* sp. nov. | BC1 | China, Sichuan Province, Dujiangyan, Chengdu | 31.14°N, 103.58°E | ZYZ-djy, Y.Z. Zhang (NS, PE) | P |
| 1 | *B. deltophylla* | CH2024-22-1 | China, Tibet, Mêdog County, Nyingchi, near Hanmi Village | 29°22'01.8"N, 95°07'05.2"E, 2226.87 m | J. Zhang, T. Gao, Y.Y. Ling (NS, PE) | M, P, C |
| 2 | *B. deltophylla* | CH2024-22-2 | China, Tibet, Mêdog County, Nyingchi, Duoxiong River | 29°24'10.1"N, 95°05'16.3"E, 2476.79 m | J. Zhang, T. Gao, Y.Y. Ling (NS, PE) | M, P, C |
| 3 | *B. deltophylla* | CH2024-22-3 | China, Tibet, Mêdog County, Nyingchi, near Rizhalu Village | 29°23'05.7"N, 95°06'07.4"E, 2364.87 m | J. Zhang, T. Gao, Y.Y. Ling (NS, PE) | M, P, C |
| 4 | *B. deltophylla* | BD1 | Nyingchi, Medog, Mo Tuo Cun,Tibet | 29.31°N, 95.36°E | ZQP-199, Z. Zhou et al. (KUN) | P |
| 5 | *B. deltophylla* | BD2 | Hanmi, Medog, Nyingchi, Tibet | 29.37°N, 95.11°E | YLZB1158, B. Xu, X.H. Xiong (photo seen) | P |
| 6 | *B. deltophylla* | BD3 | 80K, Motuo Hwy, Medog, Nyingchi, Tibet | 29.7°N, 95.52°E | ZQP-227, Z. Zhou et al. (KUN) | P |

**Table S2. Accession numbers of samples used for phylogenetic analyses of *Beesia***

| **Species** | **Isolate** | **GeneBank** |
| --- | --- | --- |
| *Beesia calthifolia* | Andrey1 | This study |
| *Beesia deltophylla* | Andrey2 | This study |
| *Beesia yangii* | Andrey3 | This study |
| *Beesia calthifolia* | BC10-1 | OQ145149.1 |
| *Beesia calthifolia* | BC11-1 | OQ145150.1 |
| *Beesia calthifolia* | BC1-2 | OQ145146.1 |
| *Beesia calthifolia* | BC12-1 | OQ145151.1 |
| *Beesia calthifolia* | BC1-3 | OQ145147.1 |
| *Beesia calthifolia* | BC1-4 | OQ145148.1 |
| *Beesia calthifolia* | BC2-1 | OQ145152.1 |
| *Beesia calthifolia* | BC4-4 | OQ145153.1 |
| *Beesia calthifolia* | BC5-3 | OQ145154.1 |
| *Beesia calthifolia* | BC6-1 | OQ145155.1 |
| *Beesia calthifolia* | BC7-3 | OQ145156.1 |
| *Beesia calthifolia* | BC8-1 | OQ145157.1 |
| *Beesia calthifolia* | BC9-1 | OQ145158.1 |
| *Beesia calthifolia* | yue-2019008 | ON342798.1 |
| *Beesia calthifolia* | yue-2019032 | ON342801.1 |
| *Beesia calthifolia* | yue-2019053 | ON342800.1 |
| *Beesia calthifolia* | yue-2019071 | ON342799.1 |
| *Beesia calthifolia* | yue-2019095 | ON342802.1 |
| *Beesia calthifolia* | ZR11-022 | NC_041531.1 |
| *Beesia calthifolia* | ZR11-022 | MK569477.1 |
| *Beesia calthifolia* |  | MK253467.1 |
| *Beesia deltophylla* | BD1-2 | NC_072729.1 |
| *Beesia deltophylla* | BD1-2 | OQ145159.1 |
| *Beesia deltophylla* | BD2-1 | OQ145160.1 |
| *Beesia deltophylla* | BD3-1 | OQ145161.1 |
| *Beesia deltophylla* |  | MZ350960.1 |
| **Outroups** |  |  |
| *Anemonopsis macrophylla* | - | NC_041527 |
| *Eranthis byunsanensis* | - | NC_066652.1 |
| *Caltha palustris* | - | NC_041532.1 |
| *Actaea cimicifuga* | - | NC_077574.1 |
| *Aquilegia kansuensis* | - | NC_058528.1 |
| *Ranunculus ternatus* | - | NC_081908.1 |
